# Clay, Zeolite and Oxide Minerals: Natural Catalytic Materials for the Ozonation of Organic Pollutants

**DOI:** 10.3390/molecules27072151

**Published:** 2022-03-26

**Authors:** Natalia Soledad Inchaurrondo, Josep Font

**Affiliations:** 1 Departamento de Ingeniería Química/Div, Catalizadores y Superficies-INTEMA-CONICET, Universidad Nacional de Mar del Plata, Mar del Plata B7606BWV, Argentina; 2 Universitat Rovira i Virgili, Departament d’Enginyeria Química, Campus Sescelades, Av. Països Catalans 26, 43007 Tarragona, Spain

**Keywords:** catalytic ozonation, natural materials, water treatment

## Abstract

Ozone has been successfully employed in water treatment due to its ability to oxidize a wide variety of refractory compounds. In order to increase the process efficiency and optimize its economy, the implementation of heterogeneous catalysts has been encouraged. In this context, the use of cheap and widely available natural materials is a promising option that would promote the utilization of ozone in a cost-effective water treatment process. This review describes the use of natural clays, zeolites and oxides as supports or active catalysts in the ozonation process, with emphasis on the structural characteristics and modifications performed in the raw natural materials; the catalytic oxidation mechanism; effect of the operating parameters and degradation efficiency outcomes. According to the information compiled, more research in realistic scenarios is needed (i.e., real wastewater matrix or continuous operation in pilot scale) in order to transfer this technology to the treatment of real wastewater streams.

## 1. Introduction

The pressure over water resources is continuously rising due to pollution, population growth and climate change. Three out of 10 people did not use a safely managed drinking water service in 2015, whereas 844 million people still lacked even a basic drinking water service [1]. In addition, Wall Street began trading water as a commodity in December 2020, and there is some uncertainty around how it will affect price and supply. Water pollution is a leading cause of death worldwide, due to the transmission of numerous debilitating diseases to populations forced to drink contaminated water [2]. Furthermore, the Intergovernmental Panel on Climate Change has recently presented a report with alarming findings, asking for immediate actions [3]. According to the report, over the 21st century, the total land area subject to drought will increase and droughts will become more frequent and severe. In this context, wastewater reclamation and reuse will be essential in coping with water scarcity and the optimization of wastewater treatment processes will be key to the safeguarding of human health and the ecological integrity of our planet.

There are numerous conventional water treatment methods (used alone or coupled) used to remove organic pollutants, such as adsorption, coagulation, flocculation, incineration, membrane separation or biological oxidation [4,5]. However, under certain circumstances, these methods present some disadvantages such as low degradation rate, poor selectivity, inhibition due to high toxicity levels, need for large space and high-energy consumption [6]. In addition, separation methods only transfer pollutants into another phase. Consequently, the development of more efficient procedures has been impelled, where the Advanced Oxidation Processes (AOPs) stand out, receiving increasing attention due to their higher degradation levels. The AOPs comprise a number of different oxidation treatments that work at nearly environmental conditions, exploiting the high reactivity of oxy-radicals, for instance, O_3_/UV, H_2_O_2_/UV, TiO_2_/UV, H_2_O_2_/O_3_, catalytic ozonation and Fenton reaction [7,8]. These methods can be used as a pretreatment to achieve the partial removal of target pollutants, reduce toxicity and improve biodegradability, before a subsequent conventional treatment [9,10]. Moreover, AOPs could function as a post-treatment method to satisfy highly strict quality control demands [10,11]. In general, these processes use expensive reagents such as H_2_O_2_ or O_3_, so their use is restricted to Chemical Oxygen Demand (COD) values lower than 5 g/L [5].

Among the AOPs, the catalytic ozonation process is a promising option for the treatment of industrial effluents due to its ability to oxidize a vast variety of refractory compounds [6]. The use of a catalyst during the ozonation process reduces the ozone requirement, minimizes bromate formation and enhances mineralization. The catalytic ozonation process has been extensively studied with a large variety of materials, mostly transition metal ions in the homogeneous version and mainly activated carbon and synthetic oxides and zeolites [6,12,13], when using a heterogeneous catalytic phase, which is highly advantageous since the catalyst can be easily recovered and reused, avoiding the secondary pollution of the metal ions. However, water treatment catalysts are often limited by their high cost. Commercial catalysts are typically more expensive than the bulk pharmaceutical aspirin (2 EUR/kg) and about the same price as vitamin C (6–8 EUR/kg) [14]. Therefore, the use of natural mineral catalysts is an interesting option since they are widely available at a low price and have shown high efficiency values in the abatement of different organic pollutants in the ozonation process. The use of natural materials could reduce the cost of the catalyst down to a few cents per kilogram. The price of minerals can vary with purity, processing, country of origin and market development. In the case of natural zeolites, values between USD 55 and 267 per metric ton have been reported, while the price of synthetic zeolite A is in the range of USD 45/kg for catalyst grade to USD 500–600/ton for detergent grade [15].

The vast majority of studies based on the synthesis of catalysts for AOPs at lab scale do not perform a cost evaluation of the material. As an exception, Martínez et al. [16] studied the synthesis of low-cost Fe/SiO_2_ catalysts for the Fenton reaction, using a simple sol-gel method and presenting a cost evaluation of the material. Although this synthesis methodology yields materials with enhanced surface and structural properties, the final cost, considering synthesis reagents as well as energy requirements, ranged between EUR 254 and 1648 per kg of pelletized sample. This cost value results much higher than that observed for natural materials.

Besides the cost of the catalyst, it is also important to evaluate the whole treatment process in terms of energy consumption and CO_2_ emissions. Therefore, several studies selected natural materials to avoid the environmental impact of complex syntheses or activation steps and the use of solvents and noble metals, in order to achieve a cleaner and more sustainable option. Within this framework, the present review describes the use of natural materials, mainly focusing on clays, zeolites and Al, Fe or Mn oxides, as catalysts in the ozonation of different organic pollutants. These types of materials (zeolites, oxides, clays or layered silicates) have been employed as catalysts for different processes, mostly as synthetic materials [6,17,18,19,20,21]. This review will only focus on the natural versions of these materials, as a cheaper and more sustainable option. The chosen natural materials present structural and surface characteristics (large surface area, presence of acid sites or hydrophobic character) that justify their use as catalysts or catalyst supports in the ozonation process. Furthermore, these materials may present a composition rich in different transition metals, which have proved to be catalytically active in AOPs. In addition, they are mostly abundant and inexpensive materials that can be obtained in high purity.

Firstly, a description of the hypothetical reaction mechanism of the catalyzed system is presented, together with its main advantages and the importance of the operating parameters chosen. Then, the characteristics and performance of the natural materials are depicted in four categories: clays, zeolites, oxides and “others”, with emphasis on degradation efficiency outcomes and reaction mechanism.

## 2. Ozonation

Ozone (O_3_) is an unstable pale blue gas that must be produced at the point of use, with penetrating smell and partial solubility in water. Due to its structure, molecular ozone can react as a dipole, an electrophilic or nucleophilic agent, during the reaction [22]. The ozonation of pollutants can proceed via two pathways: direct reaction by O_3_ molecule and indirect oxidation by HO•, which is generated through decomposition of O_3_ [23]. The direct oxidation is a selective reaction with slow reaction rate constants, typically being in the range of k = 1.0–10^6^ M^−1^ s^−1^. On the other hand, hydroxyl radicals react non-selectively and immediately (k = 10^8^–10^10^ M^−1^ s^−1^) with target molecules [11]. Normally, under acidic conditions (pH < 4) the direct pathway dominates and above pH = 10 it changes to the indirect. In ground and surface waters (pH ≅ 7) both pathways—direct and indirect—can be of importance [11]. Both oxidants, O_3_ and HO•, can attack areas of the molecules with high electron density, similarly found in unsaturated and aromatic compounds, generating intermediate products that, in later stages, can continue their oxidation until the partial or complete mineralization of the original compounds [5]. Hydroxyl radicals show a broader spectrum of oxidation, since they can also attack saturated compounds, generating complex radicals by hydrogen abstraction [5].

Ozonation is rarely used as a standalone treatment for wastewater. Complete mineralization cannot be achieved economically and the combination of ozonation with other processes is recommended, such as biological oxidation, activated carbon adsorption and membrane filtration [7,9,11,24,25,26,27].

Generally, ozonation has two specific objectives [5]:

1. Generation of BOD (biochemical oxygen demand) from DOC (dissolved organic carbon), minimizing ozone reactions with intermediate compounds. This would be the case of using ozone as a pretreatment to a biological process. In this case, the ozone concentration should be low and the process controlled by the absorption step.
2. Elimination of DOC. In this case, mineralization is required and higher O_3_ concentrations are needed.

Ozone has been used in the treatment of many types of water: ground and surface waters for drinking water use, domestic and industrial wastewaters for reuse or discharge to natural water bodies, as well as waters in swimming pools and cooling-tower systems [11].

The use of ozone to treat industrial wastewaters has encompassed the leachate from landfills or effluents from textile, pharmaceutical and chemical industries [5,6,11]. Pollutants associated with these wastewaters are mostly organic compounds refractory to biological treatments, such as humic compounds and halogenated, colored poly-aromatic compounds, endocrine-disrupting chemicals (EDCs) or pharmaceutical or personal care products (PPCPs) from the pharmaceutical and/or chemical industry, toxic or biocidal substances (e.g., pesticides), surfactants from the cosmetics industry, and COD and colored compounds from paper production [5,11]. The ozone needed can vary between 0.1 and 500 kg h^−1^, depending on wastewater volume, pollutant concentration, water matrix and the purpose of the treatment [5,11]. The ozonation technology is especially attractive for the treatment of micropollutants, which despite their relatively low concentrations (µg/L to ng/L), have significant negative effects on ecosystems and humans. Conventional mechanical–biological wastewater treatment plants are unable to completely remove most micropollutants, so ozonation and other ozonation-based processes can be used as a single-step treatment approach, as a pretreatment method followed by other conventional methods, or as a post-treatment step to achieve effluent limits [28].

## 3. Catalytic Ozonation

### 3.1. Catalytic Ozonation Advantages

The single ozonation process presents some drawbacks such as: (i) selective and/or relatively slow oxidation of some organic pollutants, which causes the accumulation of intermediate products such as carboxylic acids or aldehydes; (ii) low solubility in water that may lead to a low utilization efficiency and higher operational costs; (iii) generation of disinfection by-products (DBP), such as bromates (highly related to pH and dissolved ozone concentration) [12,13].

In order to enhance the ozonation process, several alternatives have been proposed, such as O_3_/UV, O_3_/H_2_O_2_ or the incorporation of a catalyst [22,29]. This last option has been extensively studied with diverse materials: transition metal ions in homogeneous catalytic systems (Mn^2+^, Fe^3+^, Co^2+^, Cu^2+^ and Zn^2+^) or heterogeneous catalysts such as metal oxides (MnO_2_, TiO_2_, Al_2_O_3_, FeOOH and CeO_2_), metals (Cu, Ru, Pt, Co) on supports (SiO_2_, Al_2_O_3_, TiO_2_, CeO_2_ and activated carbon), zeolites, clays, activated carbon, etc. [6,12,13].

In contrast to the single ozonation process, catalytic ozonation allows: (i) controlled generation of hydroxyl radicals or other reactive oxygen species, particularly at lower pH values; (ii) an increased mineralization level; (iii) minimization of bromate formation; (iv) increased ozone consumption efficiency [30,31,32,33,34,35,36].

### 3.2. Catalytic Ozonation Mechanism

Homogeneous catalytic ozonation includes metal/transition metal ions, such as Mn^2+^, Fe^3+^, Co^2+^, Cu^2+^ and Zn^2+^ [37,38,39,40,41] and involves mainly two pathways: (i) decomposition of O_3_ into reactive oxygen species (ROS) [40,42,43]; (ii) combination of metal ions with organic molecules to form complexes, which are then oxidized by O_3_ and/or other oxidizing species [42,44]. In spite of the good mineralization levels obtained, the addition of metal ions implies a secondary pollution, which limits the application. In heterogeneous systems, the catalyst can frequently be recovered by a simple separation step and be reused. Hence, the evaluation of different heterogeneous catalysts became a hot topic, since it is a cleaner and economical option.

There are generally three possible mechanisms of catalytic ozonation in heterogeneous systems [22]:

- chemisorption of ozone on the catalyst surface leading to the formation of active species which react with non-chemisorbed organic molecules;
- chemisorption of organic molecules (associative or dissociative) on the catalytic surface and their subsequent reaction with ozone;
- chemisorption of both ozone and organic molecules and the subsequent interaction between chemisorbed species.

Depending on the catalyst chosen, target pollutant and operating conditions, these interactions could lead to: (i) formation of reactive oxygen species (ROS), e.g., hydroxyl radicals (HO•) and superoxide radicals (O_2_•^−^) [45,46,47,48,49,50]; (ii) formation of a surface complex with the target compound more reactive towards O_3_ or other reactive oxygen species [31,42,51,52,53]; (iii) accumulation of the reagents in the vicinity of the catalyst surface, increasing the contact probability and subsequent oxidation with O_3_ or other reactive oxygen species [54,55,56,57]; (iv) enhancement of ozone dissolution and mass transfer due to O_3_ adsorption on the catalyst surface [58,59].

In order to elucidate the catalytic reaction mechanism, the authors implemented different strategies. For example, it is difficult to prove the adsorption of ozone on solid surfaces in an aqueous medium since almost all adsorptive centers of relevance to catalytic processes (i.e., surface hydroxyl sites in oxides) reveal high affinity towards water [12,60]. To deal with this issue, in situ attenuated total reflection FTIR (ATR-FTIR) spectroscopy was employed to study the interaction between ozone and oxide catalyst surfaces, using heavy water (D_2_O) instead of H_2_O, to distinguish it from the bulk OH in catalysts [60,61,62].

A common technique employed to evaluate the presence of radicals in the reaction pathway is the electron spin resonance (ESR) method. In this method, free radicals are trapped by radical trapping reagents and form stable reaction products named ‘‘spin adducts’’, which can be directly measured by (EPR) spectroscopy [57,63,64,65,66,67].

In addition, several studies use different scavenging compounds in order to evaluate the intervention of different reactive oxygen species in the catalytic mechanism: tert-butanol (TBA), HCO_3_^−^ or NaHSO_3_ for HO•; p-benzoquinones and superoxide dismutase for O_2_•; sodium azide (NaN_3_) for ^1^O_2_; catalase for H_2_O_2_ [48,49,57,67,68]. However, there are several issues to be considered regarding the analysis performed around the scavenging effect of the mentioned compounds in the abatement of pollutants. For example, TBA is the most used hydroxyl radical scavenger, since it reduces the ozone consumption rate by a factor of seven with concentrations around 50 µM [11]. However, it contains hydrophilic and hydrophobic parts, which could influence the surface characteristics of the reaction solution, decreasing the size of O_3_ gas bubbles and enhancing mass transfer and consequently, the pollutant’s oxidation [69,70,71].

In order to evaluate the possibility of a radical-based mechanism, many authors choose target pollutants that are mainly oxidized by radicals and not O_3_: oxalic, pyruvic, benzoic, p-chlorobenzoic and 1,3,6-naphtalenetrisulfonic acids, nitro- and chlorinated aromatics [38,42,45,72,73,74,75].

Guo et al. [50] presented an interesting study comparing the conventional scavenger approach (2 mM TBA or 0.63 mM para-benzoquinone) against a low concentration probe approach (0.64 μM p-chlorobenzoic acid and 0.42 μM chloroform) for the identification of reactive oxygen species (ROS) in catalytic ozonation. The authors noted that because ^1^O_2_ exhibits similar reactive selectivity towards organics as O_3_, the effect of ^1^O_2_ on enhancing ozone-resistant pollutant’s abatement is typically negligible in catalytic ozonation. According to their results, a high concentration of TBA interrupts the radical chain reactions that promotes the generation of various ROS, not only HO•, but also O_2_^•–^, O_3_^•–^, etc. Thus, rather than just acting as a HO• scavenger, the added TBA can significantly change the ozone chemistry [50]. Therefore, TBA is a useful technique to investigate the reaction mechanism of molecular O_3_, since the interferences of radical ROS can be largely precluded. In regard to p-BQ, the authors highlighted that it is also highly reactive towards O_3_, so the decrease in the pollutant’s abatement efficiency should not be solely attributed to the scavenging of HO• and O_2_^•–^. Finally, according to the authors, the conventional scavenger approach could not realistically reflect the role of the different ROS (HO• and O_2_^•–^), so they proposed spiking low-concentration ROS probes to measure ROS exposures during catalytic ozonation, which did not noticeably influence the reaction mechanism. Then, the pollutant’s abatement was satisfactorily simulated using chemical kinetic models and the respective contribution of various ROS could be quantitatively evaluated.

Additionally, the identification of active sites is key to understanding the reaction mechanism. Phosphate, carbonate and bicarbonate have been employed to identify active sites, due to their high affinity towards Lewis acid sites and capacity to block them [76]. The activity and efficiency of the catalyst depend on the nature of the active sites, textural properties such as surface area, porosity and mechanical strength. Therefore, characterization of the catalyst is highly important to comprehend the reaction mechanism and enhance its activity, which implies the determination of functional groups; crystalline structure; amount, density and dispersion of the active sites in the support; surface charge and changes in the catalyst after use. Some of the most common characterization techniques are: X-ray diffraction analysis (XRD), temperature-programmed desorption (TPD), temperature-programmed reduction (TPR), thermogravimetric analysis (TGA), N_2_ adsorption–desorption (BET surface area and porosity), scanning electron microscopy (SEM), transmission electron microscopy (TEM), energy dispersive X-ray spectroscopy (EDS, EDX), X-ray fluorescence (XRF), X-ray photoelectron spectroscopy (XPS), UV–Vis diffuse reflectance spectra (UV–Vis DRs), Fourier transform infrared spectroscopy (FTIR), Raman spectroscopy [77].

Besides the identification of active sites and reactive species involved in the oxidation mechanism, well-performed activity tests are mandatory in order to understand the real contribution of the material. Then, reaction tests must be performed taking into account:

(i) Adsorption of the target pollutant and reaction intermediates: The target pollutant or intermediate reaction products may be adsorbed on the catalyst surface, generating an additional decay in the concentration of the compound, TOC (total organic carbon) or DOC, which could be misunderstood as catalytic activity. Therefore, the nature of the model molecule and its affinity towards the catalyst surface is of the utmost importance when evaluating catalytic activity. In addition, the adsorption of organic or inorganic species could block the actives sites. For example, Qi et al. [78] studied the catalyzed ozonation of 2-methylisoborneol (MIB), using different aluminum oxides: γ-AlOOH (HAO) and γ-Al_2_O_3_ (RAO). Both HAO and RAO could enhance ozone decomposition to generate hydroxyl radicals in the absence of MIB. However, the MIB adsorption capability of HAO was higher than that of RAO. Then, the adsorption of MIB on surface hydroxyl groups in HAO reduced the number of active sites participating in the ozonation reaction, inhibiting its catalytic effect.
(ii) pH evolution during the reaction: Higher pH values promote O_3_ decomposition and generation of hydroxyl radicals. For example, Nawrocki and Fijołek [79] studied the catalytic activity of alumina and observed that sodium, the main contaminant in alumina, causes a pH increase after the introduction of the oxide in water. Then, ozone decomposition was attributed to the increase in pH and not to the true catalytic activity of the oxide.
(iii) Leaching of active species and their contribution to the reaction: Yang et al. [80] determined the metal leaching from several solid catalysts (copper- and silver-oxide-based catalysts) and investigated the influence of the leached ions on the mineralization of two model compounds (oxalate and nitrobenzene). The homogeneous catalytic effect was found to be the dominant mechanism for the degradation of the model compounds under the chosen experimental conditions. The study aimed to draw attention to this important issue, since the homogeneous catalytic contribution could occur at very low concentrations, and just reporting the metal load over the solid catalyst before and after the reaction is insufficient. Moreover, the study presented by Inchaurrondo et al. [81] reported that the activity of the natural aluminosilicate, Montanit300^®^, was associated to the leached Mn, promoted by the interaction between the catalyst surface and carboxylic acids in solution. It is important to highlight that the homogeneous contribution was observed even at very low Mn concentrations (0.0074–0.066 mg/L). Then, the small traces of Mn present in the solid catalyst were key to the outstanding activity observed.

Considering what was described above, the total effect of catalytic ozonation must be higher than the combined effect of adsorption and ozonation alone, at the same pH, and must take into account the contribution of leached species.

### 3.3. Main Operating Parameters in Catalytic Ozonation

#### 3.3.1. Ozone Dose

The O_3_ dose depends on the flowrate and O_3_ concentration in the gas phase. A higher O_3_ concentration and lower temperature imply a more favorable equilibrium concentration in the interface. A higher flowrate, adequate gas diffuser and vigorous agitation enhance mass transfer. All of it increases the concentration of O_3_ in the liquid bulk and consequently, the pollutant’s degradation efficiency [6,65,82]. However, further increases in flowrate and O_3_ concentration do not always result in a significant improvement in the degradation efficiency [83,84]. Therefore, the operating conditions should be optimized.

The O_3_ dosage supplied to the reaction medium will also depend on the degradation efficiency required. Then, the planning of the ozonation process involves clear objectives in order to optimize the ozone dose and minimize operating costs. This implies the study of mass transfer limitations (reactor configuration, agitation, flowrate, etc.) and kinetics of the oxidation reaction (determination of reaction rate constants). Moreover, the allowable concentration of ozone in water was suggested to be <0.05 mg/L in order to keep the fishes safe [85]. Then, it is necessary to reduce residual ozone in the treated water before releasing.

Depending on the purpose of the process, doses between 1 and 2 g O_3_/g DOC may be required [5,86]. For instance, the mineralization of industrial polluted wastewater may require ozone doses of 4 g O_3_/g DOC, which is economically unviable [5]. Then, ozonation of industrial wastewater is usually conducted after other treatments (e.g., biological reactor), which means lower ozone doses. In addition, it is important to optimize the employed ozone dose when analyzing whether a material is catalytically active or not. For example, Leitner et al. [87] observed that metal-supported catalysts could improve the ozonation process just for rather high ozone doses, especially for effluents with a high ozone demand. This means that the concentration of ozone needs to be high enough to enhance the catalytic effect.

#### 3.3.2. Water pH

Water pH is a key parameter due to its effect on the surface charge of the catalysts, organic molecules dissociation and ozone decomposition.

In basic solutions, ozone is especially unstable. This is due to the rapid formation of HO• by OH^−^ and the reaction of HO• with ozone. This reaction proceeds even in neutral solutions, where the OH^−^ concentration is very low (1 × 10^−7^ M) [23]. Then, higher pH values accelerate the decomposition of ozone generating more HO•, which enhances the degradation efficiency of organic pollutants [6,53,65]. However, this should be optimized, because excessive OH^−^ could quench the reaction [68].

Whenever ozone reacts with a compound that can be present in different protonation states, there will be a pH dependence of the rate constant [23]. When deprotonated, the electron density within a given molecule is higher, and due to the electrophilicity of the ozone molecule, the rate constant for the reaction with ozone is also higher [23,83].

In the heterogeneous catalytic process, the activity of the solid depends on the interaction between ozone and/or organic pollutants with the catalyst surface. Catalysts covered by surface hydroxyl groups will be protonated or deprotonated when the solution pH is below or above their point of zero charge pH (pH_PZC_). Several studies have proved that deprotonated or neutral surface hydroxyl groups have a strong reactivity towards ozone [65,67,68,78,88,89].

In addition, Yan et al. [53] studied the catalytic ozonation of oxalic acid with a Fe-SBA-15 catalyst and observed that the surface adsorption and formation of a Fe-SBA-15–oxalic acid complex was key to the oxidation mechanism, which was not fulfilled under high pH values. Then, for this type of interaction, pH values below pH_PZC_ resulted as more favorable.

#### 3.3.3. Water Matrix

The degradation efficiency of AOPs is the result of the combined effect of water matrix components, which may have a neutral, inhibitory or supportive effect on the reaction mechanism [28]. Organic species can serve either as inhibitors (by exposure to light, capture or adsorption) or as promoters (increasing indirect photolysis or catalyst regeneration) [28].

Natural sources of water present a complex composition, where inorganic ions, such as Cl^−^, HCO_3_^−^, CO_3_^2−^, SO_4_^2−^ and PO_4_^3−^, commonly appear. Owing to their high affinity towards basic sites (especially PO_4_^3−^), active sites in catalysts could be blocked [59,64,90]. In addition, hydroxyl radicals can be scavenged by dissolved organic matter (DOM) and some common inorganic ions in water, such as carbonate/bicarbonate [91,92]. Consequently, in real waters, only a small fraction of HO• can reach the target pollutants and as a result higher ozone doses are required to meet the water treatment goals, potentially leading to increased disinfection by-products (DBP) formation [11,93]. On the other hand, inorganic ions may act as promoters, such as the case of reactive oxygen species formation by nitrate ions and further catalysis by iron ions [28].

#### 3.3.4. Catalyst Dose

Higher catalyst loads result in more active sites for the catalytic reaction, which is directly connected to the surface area and porosity of the material and the density, dispersion and activity of the reaction sites [59,65,85]. The optimization of the catalyst by the improvement in the mentioned variables will lead to a reduction in the catalyst load required.

In a recent review on controversies and questions around catalytic ozonation in water, Nawrocki [94] emphasized the importance of using a realistic ratio of organic substrate to catalyst. In practical applications, this ratio is higher than 1; then, the author recommended to use more realistic ratios in activity tests. In addition, when using excess of catalyst, the effect of impurities is maximized and the homogeneous contribution of leached species increases.

#### 3.3.5. Temperature

There are two opposite effects caused by a temperature increment during catalytic ozonation: (i) thermal activation of the ozonation reactions and (ii) reduction in ozone solubility [91,95].

Many authors studied ozone solubility in water under different conditions and due to ozone decomposition in water, the experimental determination of the Henry’s Law constant (*H*) is not easy [96,97,98].

Biń [98] presented a critical survey of the available data on ozone solubility in different liquids. The experimental data for ozone solubility in ‘‘pure’’ water presented by previous authors (from 0 to 60 °C, although beyond 50 °C there is a lack of experimental data), was approximated to the following expression (*t* in °C, *H* dimensionless):

(1) H=a×exp(b×t)

with *a* = 1.599 ± 0.0164; *b* = 0.0473 ± 0.0004 (R^2^ = 0.99988) and standard error of estimation 0.0405.

Roth and Sullivan [96] expressed the *H* constant (atm/mole fraction) as a function of temperature and pH:

(2) $$H=3.84\times 10^{7}\times {\left[OH^{-}\right]}^{0.035}\times \exp\left(\frac{-2428}{T}\right)$$

where [*OH^−^*] = hydroxide concentration (mol/L) and *T* = temperature (K). The experimental data was obtained in the range 3.5–60 °C and pH = 0.65–10.2.

Most studies reported satisfactory results at room temperature (O_3_ solubility in water of 1.19 × 10^−2^ mol/L at 20 °C) [23,59]. In general, ozone rate constants depend on temperature, but only few studies report values in detail, showing activation energies for reactions with ozone in the range of 35–50 kJ/mol [23,99,100,101].

#### 3.3.6. Costs

The operation cost mainly depends on the energy consumption necessary for O_3_ generation. Based on the characteristics of the ozone generator, the concentration of O_3_ needed and the use of air or oxygen, the energy required for ozone production could vary between 8 and 26 kWh kg^−1^ O_3_ [102]. The use of a catalyst could increase the oxidant consumption efficiency, saving costs. However, the published works presenting the cost evaluation of catalyzed systems are scarce and most data is related to single ozonation processes.

Plumlee et al. [103] presented a very interesting study on conceptual-level unit costs and cost curves to aid the evaluation of advanced treatment trains. The authors highlighted that the cost for advanced treatments can be a significant portion of (or equal to) the cost for existing conventional treatments to which the advanced unit process must be added. However, cost savings may come from the implementation of advanced treatments to water reuse. Moreover, over time the cost of equipment continues declining, while energy efficiency improves, resulting in better perspectives for advanced treatments’ integration.

The study focused on the removal of trace organic contaminants (TOrCs, i.e., pharmaceuticals and personal care products and endocrine-disrupting compounds), mostly found in domestic wastewaters. Since conventional treatments are not designed to remove TOrCs, the authors evaluated membrane and AOP technologies, which could be added alone or together to conventional treatments as part of a treatment train for water reclamation in applications such as potable reuse. The unit processes included microfiltration or ultrafiltration membranes (MF/UF), nanofiltration or reverse osmosis membranes (NF/RO), ozone (with or without hydrogen peroxide, H_2_O_2_), ultraviolet (UV) treatment with H_2_O_2_ (UV/H_2_O_2_) and biological activated carbon (BAC). The cost curves indicated that at all plant capacities, membrane treatments represented the highest cost unit process, ozone the least, and BAC or UV/H_2_O_2_ fell in between. The authors noted that improvements in water quality may come with increased energy consumption and CO_2_ emissions, challenging the paradigm that increased effluent quality can only be environmentally beneficial. Consequently, the authors recommended the inclusion of a footprint, energy consumption and CO_2_ emissions evaluation alongside financial costs analysis, in order to assess the real positive impact of advanced treatments.

Mahamuni and Adewuyi [104] evaluated the cost (capital and operational) of different AOPs involving ultrasound. The costs were estimated based on the rate constants for different pollutants’ degradation (phenol, trichloroethylene and reactive azo dyes) and considering 90% abatement. Operation and maintenance costs included part replacement, labor, analytical methods, chemical use, and electrical requirements. The results obtained here were analyzed in another study performed by Fast et al. [105] on the critical evaluation of different AOPs (ozonation, UV irradiation, photocatalysis, Fenton reaction and integrated processes) for the oxidation of emerging pollutants. According to the authors, ozonation performed incredibly well (USD 0.32/1000 L), then Fenton (USD 3.77/1000 L), O_3_/UV (USD 10.21/1000 L), H_2_O_2_/UV (USD 81.50/1000 L) and finally TiO_2_ (USD 2285/1000 L). The study presented a ranking system, based on a comprehensive literature review, taking into account several categories, such as mechanical reliability, process reliability, flexibility, adaptability, energy consumption, climate change, eutrophication, toxicity, public acceptance, ease of use and economic feasibility. The hybrid system H_2_O_2_/O_3_ presented the highest average ranking score, whereas other processes showed comparable performance and TiO_2_ photo-catalysis received the lowest ranking.

Esplugas et al. [106] compared different AOPs (O_3_, O_3_/H_2_O_2_, UV, UV/O_3_, UV/H_2_O_2_, O_3_/UV/H_2_O_2_, Fe^2+^/H_2_O_2_ and photo-catalysis) for phenol degradation and concluded that ozonation was the most appealing choice based on degradation rate and lower costs. The study showed costs ranged from USD 1.09 per kg of phenol removed for ozonation to USD 293.1 for UV (considering 75% phenol degradation).

Ferre-Aracil et al. [107] studied the degradation of wastewater from a medium size hospital by ozone and peroxone methodologies. The authors determined kinetic constants and the chemical ozone demand based on the reactor geometry, gas hydrodynamics, mass transfer and the reaction of all oxidizable compounds. Cost evaluation was performed considering only the direct operation cost (electricity plus O_2_) for complete removal of the cytostatics (with expected additional organic matter reduction of 82%). The direct cost of the treatment resulted as below 0.3 EUR/m^3^.

Yang et al. [108] evaluated the feasibility of a hybrid process consisting of Fe (homogeneous and heterogeneous) catalytic ozonation and biodegradation (i.e., sequencing batch reactor, SBR) for the treatment of an industrial-based reverse osmosis concentrate (ROC). The Fe-based heterogeneous catalyst showed the highest potential to improve the biodegradability of ROC, although its direct COD removal efficiency was below the homogeneous. The cost evaluation was performed considering the power consumption for ozone generation, with a normalized ozone consumption of 3.6 mg O_3_/mg COD. The cost for SBR biodegradation was roughly estimated as 0.004 USD/m^3^. Then, the unit cost for ROC treatment by the coupling of catalytic ozonation and biodegradation was 0.15 USD/m^3^.

Heidari et al. [109] studied the use of limonite (raw and plasma treated) in the catalytic ozonation of the sulfasalazine antibiotic (SSZ). In this study, economic calculations were completed by assessing the electrical energy consumption, which in AOPs were evaluated as the Electrical Energy per Order (*E_EO_*), a concept provided by the International Union of Pure and Applied Chemistry (IUPAC); where *E_EO_* is the required electrical energy (in kWh) to purify contaminated water (1 m^3^) by 1 order of magnitude. The electrical energy consumption of different oxidation processes was calculated as:

(3) $$E_{EO}=\frac{P\times t\times 1000\;}{V\times log\left(\frac{TOC_{0}}{TOC_{f}}\right)}$$

where *P* is input power (kW) of the system, *t* is processing time (h), *V* is volume (L) of reaction medium; *TOC*_0_ and *TOC_f_* are initial and final total organic carbon (mg/L), respectively. The calculated *E_EO_* for the catalyzed ozonation process was significantly low compared to sole ozonation and ozonation in the presence of H_2_O_2_.

Krichevskaya et al. [110] executed energy cost calculations using data from 40 publications on pollutant degradation studies, considering phenol, glycols, methyl-tert-butyl ether (MTBE), aliphatic unsaturated compounds, humic acids and lignin as the most representative target compounds. Two oxidation processes, ozonation and Fenton reaction, were chosen as water treatment methods. The cost evaluation was based on the decrease in the target pollutant’s concentration and/or COD/TOC, available from published material, which was referred to as the energy needed for ozone generation and/or the cost of reagents. The costs of equipment, maintenance, etc., were omitted since it depends greatly on specific conditions. The summarized results from 40 publications indicated ozonation costs of: EUR 0.3–0.61 kg^−1^ of unsaturated aliphatic compounds, EUR 0.4–17 kg^−1^ of phenol, EUR 2.2–23 kg^−1^ of oxygenated compounds, EUR 0.9–3.2 kg^−1^ of lignin and as high as EUR 11 kg^−1^ for humic substances. The wide deviations on treatment costs under similar conditions was attributed to variations in ozone absorption techniques (mass transfer factors) and non-optimized ozone doses.

Gottschalk et al. [11] gathered information regarding the operational costs of ozonation for the treatment of different types of effluents. The operational cost for the production of 1 kg of ozone is typically in the range of EUR 1.5–2.0. Gottschalk et al. [11] reported operational costs for the treatment of drinking water in values from less than EUR 0.01 to 0.05 per m^3^, with similar costs in the case of WWTP effluents if disinfection and/or the removal of easily oxidizable micropollutants is the goal. Costs for industrial effluents ranged from around EUR 0.2 per m^3^ in the textile and paper industry to as much as EUR 4 for landfill leachate.

## 4. Natural Mineral Catalysts

In this section, we describe the characteristics and modifications performed in natural materials to be applied in catalytic ozonation processes, mostly centered in clays, zeolites and oxides. These materials have been employed for their intrinsic activity or as supports for the active phase. The catalytic oxidation mechanisms observed are summarized in the body of the text and the operating conditions (target pollutant, O_3_ dose, temperature, pH, catalyst) and degradation efficiency outcomes are presented in corresponding tables.

### 4.1. Clays

#### 4.1.1. Definition

According to the Association Internationale pour l’Etude des Argiles (AIPEA) and the Clay Minerals Society (CMS) [111], the term ‘clay’ refers to a naturally occurring material composed primarily of fine-grained minerals, which is generally plastic at appropriate water contents and will harden when dried or fired. Although phyllosilicates are the most common and abundant clay materials, clays may also contain other materials that impart plasticity and harden when dried or fired [111].

Other types of layered minerals that are worth mentioning are the layered alkali silicates (including kanemite, octosilicate, makatite, magadiite and kenyaite), which are a class of hydrated layered silicates composed of a silicic acid layer (with different layer thickness) and the charge compensating (neutralizing) interlayer alkaline cations [18,112]. Due to the structural characteristics (nanosheet nature with the reactivity of the surface silanol groups), these materials have been tested, raw or modified (i.e., pillared layered silicates), as adsorbents for water treatment [113,114]. Some of the layered alkali silicates such as magadiite and kenyaite are found in nature but compared with bentonite, the available amount is limited and not enough for the industrial application.

The focus in this section will be centered on phyllosilicates as the main representative clay materials used in catalytic ozonation processes.

#### 4.1.2. Structure and Properties

**Figure 1:**
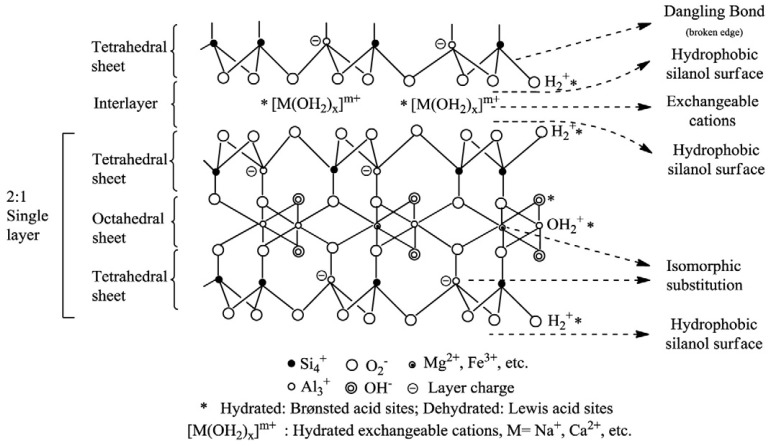
Clay’s general structure [118] (reprinted from Publication Appl. Clay Sci. 74, C. H. Zhou, J. Keeling, Fundamental and applied research on clay minerals: From climate and environment to nanotechnology, 3–9 (2013) with permission from Elsevier).

Phyllosilicates are characterized by a structure based on the presence of octahedral and tetrahedral layers with or without interlayers (Figure 1). Each tetrahedron consists of a cation coordinated to four oxygen atoms and linked to adjacent tetrahedra by sharing three corners (the basal oxygen atoms) to form an infinite two-dimensional hexagonal mesh pattern [115]. Each octahedron consists of a cation coordinated by six oxygen atoms and linked to a neighboring octahedral by sharing edges forming sheets of hexagonal or pseudohexagonal symmetry [115]. The space that can be occupied by a cation in a tetrahedral site is smaller than the space that can be occupied in an octahedral site [116]. Therefore, common tetrahedral cations are Si^4+^, Al^3+^, and Fe^3+^ and usual octahedral cations are Al^3+^, Fe^3+^, Mg^2+^ or Fe^2+^.

The central metal ion present in octahedral or tetrahedral sheets commonly suffers an isomorphous substitution (replacement by a “foreign” ion that has a different valence than those of the ions proper to the lattice), which generates the primary source of both negative and positive charges in clay minerals. For example, Mg for Al in octahedral sheets or Al for Si in tetrahedral ones, causing a negative charge in the aluminosilicate layers that is then compensated by the presence of cations within the interlayer space [21].

In addition, negative surface charge in layers may be accounted for by broken bonds at the edges of the sheets, on noncleavage surfaces [117]. This second source of charge is generally negative and pH dependent [21]. The unsatisfied charges may be balanced by the sorption of cations and anions, either specifically by chemisorption or nonspecifically by electrostatic attraction, the latter ions being easily exchangeable [117].

Clays in their natural form have been employed as adsorbent materials or catalysts, due to the presence of different types of active sites [118]. According to Johnston [119] in his study on the sorption of organic compounds on clay minerals, the six predominant active sites on clays are:

1. Neutral siloxane surface: The siloxane surface is very unreactive due to the strong bond between atoms of silicon and oxygen. However, it presents hydrophobic characteristics with interesting adsorption properties.
2. Isomorphic substitution sites: As mentioned before, the charge deficit originated from isomorphic substitution (usually Al for Si) is compensated by the presence of exchangeable cations. The location of the isomorphic substitution sites (octahedral or tetrahedral) influences the degree of polar or charged compounds’ adsorption. Isomorphic substitution in octahedral sheets creates soft Lewis base sites and in tetrahedral sheets, harder Lewis base sites. When the extent of Al for Si substitution increases, the hydrogen bonding to the charged surface by polar molecules increases.
3. Metal cations occupying cation exchange sites: Organic solutes do not replace the exchangeable metal cation, but rather coordinate directly with the cation occupying the isomorphic substitution sites.

In addition, exchangeable or structural transition metals in their upper oxidation state can interact directly with certain organic solutes. For example, a transition metal cation such as Fe^3+^ (exchangeable or structural) can act as a Lewis acid site by accepting electrons from adjacent unsaturated organic solutes, causing the reduction of the cation and generating radical organic cations on the surface. The surface Lewis acidity depends on the reduction potential of the metal cation and water content. When the content of water is high it is more difficult for organic molecules to compete for coordination positions around the hydrated metal cations.

4. Hydrophobic sites: Through the sorption of organic molecules onto the clay’s surface. For example, the exchange of inorganic cations for alkyl ammonium cations on montmorillonite.
5. Water molecules surrounding exchangeable cations: The polarization of water molecules surrounding exchangeable cations or coordinated cations at broken edges (see point 6) generates Brönsted acid sites (proton donors). The strength of acidity depends on the nature of the exchangeable cation and amount of water. The presence of cations with higher charge and reduced size results in higher acidity.
6. Broken edge sites and exposed surface silanol and aluminol groups: These are surface hydroxyl groups located on the broken edges of clay minerals, which can form inner-sphere complexes with metal species, hydrogen bond to molecules accumulated at the interface, undergo replacement reactions with deuterium, tritium, and F^−^ or be influenced by inorganic or organic cations through electrostatic interactions. These structural hydroxyl groups are among the most abundant and reactive active sites found on particles in soil. As the particle size decreases, the contribution of these sites to the overall reactivity increases.

#### 4.1.3. Modification of Natural Clays

The characteristics of clays can be enhanced though different simple techniques, such as:

(i) Ion-exchange reaction: The cation exchange capacity of clays is the most important parameter, which depends on the layer charge density. Cations present in the interlayer space of clays can be exchanged for the desired catalytic ion. Moreover, hydrophilic clays can be changed into hydrophobic clays, for example, with the exchange of inorganic cations for alkyl ammonium cations.

The larger the number of cations in the interlayer, the more strongly the platelets are bound together, limiting the access of the reactants [21]. Incorporation of heavier metal cations displaying high solvation capacity is known to generate Brønsted and Lewis acid sites. The Brønsted acidity originates from the dissociation of the intercalated water molecules coordinated to cations [120].

(ii) Pillared interlayered clays (PILC): The interlamelar space in clays is generally not accessible to all substrates due to the strong electrostatic interaction between sheets and charge balancing cations. Therefore, modification through the introduction of large cations (“pillars”) between the sheets followed by calcination (the polycations are converted into the corresponding metal oxide clusters), increases porosity and surface area. In addition, the pillars made by a combination of Al with other metals (i.e., iron) introduce or constitute additional catalytic sites [121].
(iii) Acid activation: The treatment with acids causes ion exchange reactions with H^+^, breakdown of layers causing more broken edge bonds and a reduction in particle size, and a selective leaching of central atoms in tetrahedral and octahedral sheets. In general, the acid treatment causes an increment in the amount of acid sites and surface area of the materials [21]. In addition, drastic acid treatments may cause dealumination (higher Si:Al ratio), increasing the hydrophobic characteristics of the material.
(iv) Calcination: Purification of the materials is often performed through controlled calcination up to 400–500 °C, in order to remove carbonates and organic impurities.

Heating up to 200–300 °C decreases Brönsted acidity and increases Lewis acidity. At higher temperatures (450 °C and above) complete dihydroxylation of the aluminosilicate lattice converts Brönsted acid sites into Lewis sites [120].

#### 4.1.4. Application of Clays in Ozonation Processes

Several authors have reported the use of raw and modified clays in the catalytic ozonation of different organic pollutants at a laboratory scale. The operating conditions and results obtained in some of these studies are briefly presented in Table 1.

Boudissa et al. [122] studied the catalytic ozonation of different cationic and anionic dyes in the presence of raw and modified clays: ion-exchanged montmorillonite (NaMt and Fe(II)Mt), crude bentonite and acid-activated counterparts (HMt: H_2_SO_4_ 5 M 80 °C, different treatment times from 1 to 24 h). Results from CO_2_ and water TPD measurements allowed to connect the acid–base properties and hydrophilic character of the materials with the behavior of dyes in water (dissociation at different pH values) and removal capacity. The higher catalytic effectiveness was mostly related to dye adsorption. Electrostatic interactions prevailed for certain dyes on certain catalysts, which was related to the point of zero charge and surface charge of the materials and dissociation constants of the organic molecules. On Fe(II)Mt, the hydrophobic interaction, cation exchange and Fe^2+^ mobility were key to explain the catalytic activity. In the case of HMt, acid activation increased the hydrophobic character of bentonite (higher Si/Al ratio), promoting adsorption and catalytic activity.

Chung et al. [63] used thermally treated clay from tidal flat sediments. The material was purified through washes with H_2_O_2_ solution and then it was calcined at different temperatures: 300, 500 and 700 °C, during 2 h. The treated clays were tested in the catalytic ozonation of perchloroethylene (PCE) obtaining high conversions (93.9%) based on a hydroxyl radical mechanism, validated by EPR spectroscopy characterization.

Gao et al. [58] used a combined system of MnO_2_ and kaolin to enhance the ozonation of the cationic dye, methylene blue. The presence of kaolin significantly improved ozone mass transfer. Ozone was accumulated on the surface of kaolin and then reacted with MnO_2_ yielding free radical species.

Liu et al. [123] prepared a CuFe_2_O_4_ nanocomposite loaded onto natural sepiolite (CuFe_2_O_4_/SEP) by the citrate sol-gel method. The material was tested in the catalytic ozonation of quinoline. The authors chose the magnetic spinel ferrite nanoparticle cuprospinel, since it has more oxygen vacancies and surface hydroxyl groups than CuO or Fe_2_O_3_. According to the study, the surface hydroxyl groups and Lewis acid sites on CuFe_2_O_4_/SEP reacted with O_3_ generating the hydroxyl and superoxide radicals responsible for quinoline degradation. Sepiolite not only played an important role as a support, avoiding the agglomeration of the nanoparticles (dispersing the active phase), but also promoted the migration of oxygen species from the active sites to oxygen vacancies. Adsorption and oxygen stripping had a negligible effect on TOC removal and the efficiency of quinoline mineralization in the catalytic ozonation process was 5.4 times higher than that of the un-catalyzed system. Five-cycle successive tests suggested that the material was recyclable and, hence, stable.

**Figure 2:**
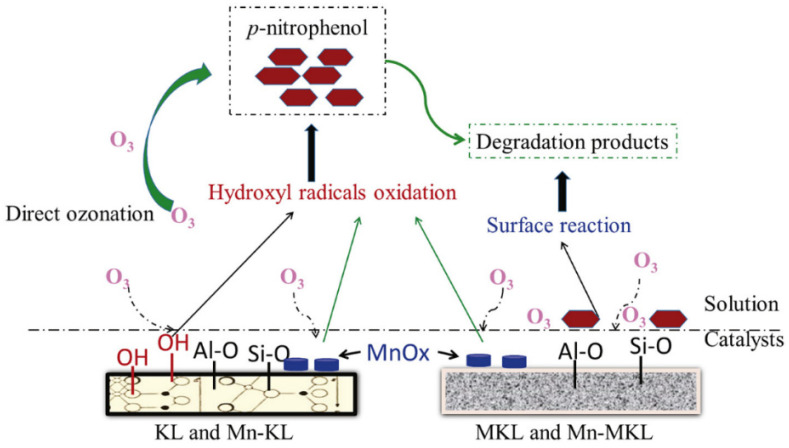
Proposed ozonation mechanism of p-nitrophenol in catalytic ozonation treatments using kaolinite-based catalysts [124] (reprinted from Publication Appl. Clay Sci. 175, W. Ma, J. Hu, B. A. Yoza, Q. Wang, X. Zhang, Q. X. Li, S. Guo, C. Chen, Kaolinite based catalysts for efficient ozonation of recalcitrant organic chemicals in water, 159–168 (2019) with permission from Elsevier).

Ma et al. [124] studied the ozonation of p-nitrophenol using kaolin (KL), metakaolin (MKL) and Mn oxides supported on KL and MKL. Metakaolinite is obtained from the calcination of kaolinite and both materials are constructed by silica and alumina tetrahedron but have different structures. KL presents a structure based on layer phyllosilicate crystal and MKL, amorphous silicon aluminum. The authors focused on testing materials with identical components, which may have contrasting behaviors due to structural differences, in concordance with other authors [78,125]. All the materials enhanced the ozonation process though different mechanisms (Figure 2). In the case of MKL, the introduction of NaHCO_3_ (radical scavenger) did not change the TOC removal efficiency, indicating the absence of radicals. Therefore, a mechanism based on the activation of adsorbed ozone and pollutants was suggested, promoting their reaction. In the case of KL- and Mn-supported materials, a mechanism based on hydroxyl radicals was detected. Compared to MKL, the increased catalytic activity of KL was related to additional surface OH groups. The presence of Mn oxides promoted the catalytic activities of both Mn-KL and Mn-MKL, with better results in the case of Mn-KL, due to the formation of favorable aggregated Mn oxide species (Mn^2+^, Mn^3+^, and Mn^4+^), as well as the additional surface OH groups. The reusability of the materials was studied and no remarkable changes in TOC removal was observed after five uses, although Mn leaching was detected.

**Table 1:** Clay minerals as catalysts in the ozonation of organic pollutants.

Catalyst	Pollutant	Operating Conditions	Reaction Outcomes (X: Percentage Conversion)	Ref.
Crude bentonite (B), acid activated montmorillonite (HMt) and ion exchanged montmorillonite (NaMt, Fe(II)Mt)	Cationic dyes (methylene blue and methyl green) and Anionic dyes (methyl orange and methyl-thymol blue)	Semibatch reactor, O_3_ dose 600 mg/h, 22 °C, 20 mL of 10^−4^ M dye, catalyst load 40 mg, 5 min.	Dye conversion yields: B: 94–100%, NaMt: 72–96%, Fe(II)Mt: 95–96%, HMt-1:99–100%. The catalyst addition accelerated changes in relative absorbances of most UV-vis bands below 350 nm.	[122]
Thermal treated clay from tidal flat sediments	Perchloroethylene (PCE)	Batch reactor, O_3_ saturated water (5.8 ppm), 20 °C, 50 mL of 0.03 mM PCE, 0.8 wt.% clay, 10 min.	PCE Conversion: O_3_ alone: 60.6%, O_3_ + clay treated at 700 °C: 93.9%.	[63]
Kaolin plus MnO_2_	Methylene blue (MB)	Bubbled column semibatch reactor, O_3_ dose 2.5 g/h, room T, 0.2 L of 0.3 g/L MB, 10 g kaolin and 5 g MnO_2_, 5 min, pH_0_ 11.	COD Conversion: MnO_2_ + O_3_: 20%, kaolin+O_3_: 40%, MnO_2_ + O_3_ + kaolin: 80%.	[58]
CuFe_2_O_4_ nanoparticles supported on sepiolite (SEP)	Quinoline (QN)	Semibatch reactor, gas flow rate 1 L/min, room T, 0.5 L of 50 mg/L QN, catalyst load 1 g/L, 30 min, pH_0_ 6.8.	TOC Conversion: O_3_ alone: 16.8%, SEP + O_3_: 44.1%, CuFe_2_O_4_ + O_3_: 55.8%, CuFe_2_O_4_/SEP + O_3_: 90.3%. Homogeneous contribution: 23.7%. Leaching: 0.41 mg/L Cu and 5.2 µg/L Fe. Negligible adsorption.	[123]
Kaolinite (KL), Metakaolinite (MKL) and Mn oxides supported on KL (Mn-KL and Mn-MKL, 3% Mn loading)	p-Nitrophenol (NP)	Semibatch column reactor 0.25 L, gas flow rate 0.5 L/min, 30 °C, 0.1 L of 300 mg/L NP, catalyst load 0.5 g, 30 min, pH_0_ 5.2.	TOC Conversion: Adsorption: 1–3%, O_3_ alone: 32%, KL+O_3_: 46%, MnKL + O_3_: 66%, MKL + O_3_: 41%, MnMKL + O_3_: 54%. Manganese leaching: 5.8% MnKL and 6.5% MnMKL.	[124]
Ion exchanged montmorillonite (NaMt, FeMt; CoMt; NiMt; CuMt)	Oxalic acid (OA)	Semibatch reactor, O_2_+O_3_ mixture throughput 5 mg/min, 25 °C, 0.5 L of 2.5 × 10^−3^ M OA, catalyst load 1 g, 180 min.	OA Conversion: O_3_ alone: 22.5%, Crude Mt + O_3_: 69%, NaMt + O_3_: 70.4%, CuMt + O_3_: 78.6%, CoMt + O_3_: 80.1%, NiMt+O_3_: 83.1%, FeMt + O_3_: 95.2%.	[52]
Ion exchanged montmorillonite (NaMt, FeMt; CoMt; NiMt; CuMt)	Oxalic acid (OA)	Semibatch reactor, O_3_ stream 6 mg/min, 25 °C, 10^−3^ M OA, catalyst load 2 g/L, pH 2.8, 60 min.	OA conversion: O_3_ alone: 1–2%, Crude Mt + O_3_: 5–6%, NaMt+O_3_: 10%, Fe(III)Mt + O_3_: 48%, Cu(II)Mt + O_3_: 70%, Co(II)Mt + O_3_: 95%, Ni(II)Mt + O_3_: 83%, Fe(II)Mt + O_3_: 100%.	[95]
Ion exchanged montmorillonite (NaMt, FeMt; CoMt; NiMt; CuMt)	Sulfamethoxazole (SMX)	Semibatch reactor, O_3_ feed 6 mg/min, 3 × 10^−4^ M SMX, catalyst load 1.91 g/L, pH 2.88, 20 min.	COD Conversion: NaMt + O_3_: 84%, FeMt + O_3_: 98%, CuMt + O_3_: 92%, CoMt + O_3_: 97%, NiMt + O_3_: 95%.	[120]
Fe-pillared clay	Indigo carmine (IC)	Semibatch bubble column reactor, gas flow rate 0.045 L/min, average O_3_ production 0.005 g/L, 1000 mg/L IC, 19 °C, catalyst load 0.1% *w*/*w*, pH 3, 10 min.	IC conversion: Adsorption: 2.9%, O_3_ alone: 25%, bentonite + O_3_: 30%, Fe particles: 40%, Fe-pillared clay + O_3_: 98%.	[126]
Montmorillonite (K10) and Al-Fe modified Montmorillonite (AFK 10)	Methylene blue (MB) and Malachite green (MG)	Semibatch reactor 0.06 L, O_3_ dose 0.5 g/h, 30 °C, 5 × 10^−5^ M dye, catalyst load 5 mg/L, 40 s.	Dye conversion: Adsorption: MB/K10: 78%, MG/K10: 51%, MB/AFK10: 24%, MG/AFK10: 56%. O_3_ alone: MG: 46%, MB: 17%. Catalytic ozonation: MB/K10: 59%, MG/K10: 70%, MB/AFK10: 99%, MG/AFK10: 88%.	[127]
Sepiolite (SEP) and Zero valent iron on sepiolite (ZVI/SEP)	Caffeine	Semibatch reactor 0.25 L, gas flow rate 6 mg/min ([O_3_]_aqueous_ = 4 mg/L at t = 0), 25 °C, 10 mg/L caffeine, catalyst load 1 g/L, 60 min, pH 6.	TOC Conversion: O_3_ alone: 17.5%, SEP+O_3_: 30%, ZVI/SEP + O_3_: 41.1%. Caffeine adsorption 76%.	[128]
Kaolinite (KAO) from sulfide flotation wastewater	O-isopropyl-N-ethylthionocarbamate (IPETC)	Semibatch reactor 2.5 L, gas flow rate 1.67 L/min, O_3_ dose 2.065 mg/(min L), 20 °C, 2 L of 100 mg/L IPETC, catalyst load 0.5 g/L, 180 min, pH_0_ 10.	IPETC Conversion (60 min): O_3_ alone: 84.81%, KAO + O_3_: 98.31%, adsorption: 4.96%. TOC Conversion (180 min): O_3_ alone: 12.47%, KAO + O_3_: 29.01%, adsorption: 2.58%.	[129]
Nanovermiculite loaded with Fe^2+^	Sanitary landfill leachate	Semibatch rotating bed reactor 1.6 L, rotation speed 915 rpm, gas flow rate 3.9 L/min, O_3_ generator capacity: 34 g/h at 5 L/min, 23 °C, 0.2 L of 860 mg/L COD, catalyst load 0.05 g, 60 min, pH 5.8.	COD Conversion: Catalytic ozonation: 15.9%, adsorption: 1.7%. Color removal: 42.4%.	[130]
Raw sepiolite (SEP), Modified sepiolite (MOD-SEP), Mn-loaded modified sepiolite (Mn-SEP)	Regenerated papermaking wastewater	Semibatch reactor 1.6 L, gas flow rate 0.4 L/min, O_3_ 20 mg/L in gas phase, room T, 1 L of 140–200 mg/L COD, catalyst load 0.8 g, 30 min, pH_0_ 8.	COD Conversion: O_3_ alone: 34.8%, SEP + O_3_: 50.3%, MOD-SEP + O_3_: 62.7%, Mn-SEP + O_3_: 73.4%. Mn leaching < 0.2 mg/L.	[131]

Several studies focused on using ion-exchanged montmorillonite to catalyze the ozonation of different pollutants with outstanding results [52,95,120]. Azzouz et al. [52] studied the ozonation of a very recalcitrant contaminant, oxalic acid, using Fe, Co, Ni, and Cu exchanged montmorillonite (FeMt, CoMt, NiMt, CuMt). The presence of these materials enhanced the oxidation process through a mechanism based on the adsorption of oxalic acid and molecular ozone on the clay surface. The magnitude of the interaction between the catalyst surface and the reactants was correlated to the global acid character of the materials, which was defined as the overall amount of both physically and chemically adsorbed butylamine (titration in aprotic solvent method). The reactants’ adsorption via acid–base interactions with the exchangeable cations was involved in the reaction mechanism. Unless completely released in the liquid media, these cations combined with oxalate anions previously adsorbed or merely attracted to the clay surface and, then, metal(II)-oxalate complexes accumulated in the vicinity of the clay surface. This process was deeply dependent on pH and clay concentration. It was observed that low pH values favored the oxidation reaction by accentuating the concentration of these cations, and, thereby, adsorption of oxalates. In addition, van der Waals interactions and coulombic attraction between oxalic acid and anionic sites were also expected to contribute to oxalate adsorption. Moreover, at low pH silanol and aluminol terminal groups are protonated, and may exert coulombic attraction upon oxygen atoms of the ozone molecule, enhancing adsorption. A decay in activity was observed at high pH levels, related to the progressive removal of the exchangeable cations. Under this condition, eventual regeneration with fresh Fe(II) is needed. FeMt showed the highest degradation levels due to its moderate surface acidity, while pronounced surface acidity in NiMt and CuMt resulted in lower oxalic acid conversions. The authors explained that excessively acidic surfaces might hinder oxalic acid adsorption, by attenuating or shading the base character of the oxygen atoms surrounding the exchangeable cation that act as adsorption sites. The catalytic activity of the crude clay mineral was quite remarkable and similar to NaMt. The latter was connected to compensating phenomena: (1) the disappearance in NaMt of the catalytic activity promoted by exchangeable cations; (2) the improvement in the surface area in NaMt (almost double). In addition, clay dispersion was studied and connected to the relatively lower effectiveness of NaMt. This material showed a larger flocculation capacity, related to the specific net charge of the Na cations. Moreover, high catalyst loads were detrimental, because they favor lamella–lamella interactions, promoting coagulation–flocculation and finally reducing the available surface area. In a subsequent work, Shahidi et al. [95] delved into the study of the catalytic ozonation of oxalic acid with ion-exchanged montmorillonite. It was observed that the role of montmorillonite not only involved the catalytic effect of the exchangeable cations but also the contribution of adsorption on the clay mineral surface. Total removal of oxalic acid was achieved at pH 2.87 with 1.88 g/L of Co(II)Mt, or at pH 2.88 with 1.91 g/L of Fe(II)Mt. Under these conditions, the mechanism was supposed to be heterogeneous, but the homogenous contribution from free metal cations resulting from partial ion exchange was not discarded. Higher pH values and catalyst loads increased clay compaction, reducing cation mobility and reactants adsorption.

Shahidi et al. [120] also studied the use of these low-cost clay-based materials (NaMt, FeMt; CoMt; NiMt; CuMt) for the degradation of an emerging pollutant, sulfamethoxazole (SMX). All the materials tested were able to achieve high mineralization levels. Fe(II)Mt allowed the total mineralization of all organic substrates in less than 40 min. Kinetic calculations revealed that ozonation starts in the bulk solution, while adsorption is progressively enhanced in time by the appearance of intermediates.

Bernal et al. [126] studied the ozonation of indigo carmine with Fe-pillared clay. There are several studies regarding the use of this type of material in other AOPs (mostly Fenton reaction) [8,121], but in catalytic ozonation processes they are scarce. In this study, the addition of small amounts of clay substantially enhanced the pollutant’s degradation level. The authors suggested a reaction mechanism based on hydroxyl radicals enhanced by the presence of ferrous species in the catalyst. No stability tests were performed but the authors’ suggested reuse is plausible, since neither leaching nor Fe oxidation state changes were detected.

Mirilă et al. [127] studied the catalytic ozonation of two organic dyes, Methylene blue (MB) and Malachite green (MG), with montmorillonite (K10) and its Al-Fe modified counterpart (AFK10) (impregnation by a solution of Fe^2+^(FeCl_2_·4H_2_O) and Al^3+^ (AlCl_3_·6H_2_O)). The authors studied separately the adsorption, non-catalyzed and catalyzed ozonation processes in order to elucidate the role of the materials. The catalytic enhance was based on the reactants’ adsorption on the clay’s surface, involving specific acid-base, electrostatic and/or hydrophilic/organophilic interactions according to the dye molecule and catalyst surface properties. The ozonation of adsorbed dyes prevailed at the expense of that occurring in the bulk solution. According to characterization results (CO_2_ and water TPD measurements), AF-K10 presented higher basicity (of weak strength) due to the incorporation of amphoteric to slightly basic Al-OH groups and K10 showed a higher but weak hydrophilic character. Adsorption turned out to be much more favorable for MB on K10 and slightly higher for MG on AF-K10. In all cases, the mere addition of the catalyst produced a marked increase in the yield of dye removal, showing a far more pronounced effect in the case of AF-K10, where hydrophobic interactions prevailed, overcoming the low gas solubility in aqueous media.

Savun-Hekimoğlu et al. [128] studied the use of sepiolite, a low-cost natural clay mineral found abundantly in Anatolia, as a catalyst in the ozonation of caffeine. The material was tested raw (SEP) and modified by the immobilization of zero-valent iron (ZVI/SEP), using sonolytic intercalation. The presence of both, the raw and modified catalyst, enhanced the degradation rate and mineralization levels. The activity of SEP was attributed to its surface rich in hydroxyl groups. The immobilization of zero-valent iron (ZVI) increased the surface area and the high reactivity of Fe^0^ at the surface of the mineral, initiated strong redox reactions with the adsorbed species, with the likely generation of different reactive oxygen species. A reaction mechanism was proposed; however, no specific tests were performed to verify the presence of different radical species. The integration of the process with high-frequency ultrasound (US) further improved the reaction outcomes. The ZVI/SEP sample was stable, with only 5, 6, 8 and 9% loss in activity upon each consecutive cycle and no leaching was detected.

Fu et al. [129] studied the catalytic ozonation of O-isopropyl-N-ethylthionocarbamate (IPETC), which is a flotation reagent, from sulfide flotation wastewaters. The authors used a coexisting Kaol as catalyst, which is a clay mineral widely present in flotation wastewaters as suspended solids. The material was mainly composed of Al_2_O_3_ (46.04%), SiO_2_ (52.87%), Fe_2_O_3_ (0.18%) and TiO_2_ (0.11%), with a specific surface area of 12.07 m^2^/g. FTIR characterization of the solid sample was performed to identify surface OH groups, to which the activity of the material is usually attributed. The presence of the catalyst enhanced the abatement and mineralization of the pollutant. The catalyzed system showed higher mineralization extents of C, S and N elements and conductivity levels. The parametric study of pH and catalyst load indicated better outcomes with a higher catalyst dosage and basic pH values (sulfide flotation always operates at pulp pH of 9–12). The presence of Kaol decreased the equilibrium ozone concentration at any initial pH, since the material increased the oxidant decomposition. Tests performed with TBA confirmed the formation of HO•, which could be generated from both the catalytic decomposition of ozone on the Kaol surface and bulk ozone decomposition initiated by OH^−^ anions. Moreover, the ratio of the HO• exposure to the O_3_ exposure (*R_ct_*) was evaluated at pH 10. pCBA was used as a HO• probe. The *R_ct_* value was determined according to Equations (4) and (5) by measuring the concentrations of *p*CBA and aqueous ozone during the ozonation:

(4) $$R_{ct}=\frac{\int_{t}^{0}\left[•\mathrm{OH}\right]\mathrm{dt}}{\int_{t}^{0}\left[O_{3}\right]\mathrm{dt}}$$

(5) $$R_{ct}=\frac{ln\left\{\frac{{\left[pCBA\right]}_{0}}{{\left[pCBA\right]}_{t}}\right\}}{k_{pCBA/•\mathrm{OH}}\int_{0}^{t}\left[O_{3}\right]\mathrm{dt}}\;$$

The presence of natural Kaol significantly improved the yields of HO•s (*R_ct_*) in pure water (increased HO• formation and ozone mass transfer).

In addition, adsorption of by-products was studied by XPS, which revealed that the by-products adsorbed on the Kaol surface changed during the catalytic reaction. Then, surface oxidation reactions by active species also contributed to IPETC removal and mineralization.

Moreira Braga et al. [130] used modified nano-vermiculite in a rotating bed reactor for the catalytic ozonation of sanitary landfill leachate. The rotation of the bed, initial pH, and ozone flow were analyzed through experimental design represented by the central composite design (CCD) and later optimized through the Normal Boundary Intersection (NBI) algorithm. The vermiculite was thermally treated (600 °C) and loaded with Fe by impregnation with an ethanol solution of iron nitrate. XRD and FTIR characterization showed the expansion of the material through the release of water molecules. After impregnation, the Fe content rose to 12.5% and surface area increased from 5.87 to 18.08 m^2^/g. The catalytic process allowed increasing the removal of color and COD above 50%. According to the authors, the activity of the catalyst was attributed to the availability of Fe (II) ions that may react with molecular ozone yielding hydrogen peroxide and hydroxyl radicals, especially under acidic pH. At basic pH values, ferric oxyhydroxides precipitate, decreasing the concentration of available Fe (II) ions in the medium. Nevertheless, the presence of OH− enhances ozone decomposition into hydroxyl radicals. Stability tests or Fe leaching measurements were not performed.

Cheng et al. [131] tested modified sepiolite in the catalytic ozonation of regenerated papermaking wastewater (after biological treatment). Briefly, the authors treated the material with HNO_3_ (modified sepiolite), loaded with Mn(NO_3_)_2_ and then calcined at 350 °C. The specific surface area of modified sepiolite (421.8 m^2^/g) and Mn/sepiolite (412.3 m^2^/g) were both more than three times higher than that of the natural sepiolite (124.6 m^2^/g). According to XRF measurements, the manganese content significantly increased (3.84 wt.%) and was well dispersed on the catalyst surface (XRD characterization). The raw and modified samples enhanced the degradation of the pollutants. The Mn/sepiolite sample showed stability over five runs (COD removal efficiency remained higher than 72.9%). However, Mn leaching was detected (<0.2 mg/L), but at a lower concentration than discharge limits. The authors attributed the activity of the catalyst to the generation of hydroxyl radicals, but it was not verified.

### 4.2. Zeolites

#### 4.2.1. Definition

The Subcommittee on zeolites of the International Mineralogical Association, Commission on new Minerals and Mineral names arrived at the following definition of zeolite:

“A zeolite mineral is a crystalline substance with a structure characterized by a framework of linked tetrahedra, each consisting of four O atoms surrounding a cation. This framework contains open cavities in the form of channels and cages. These are usually occupied by H_2_O molecules and extra-framework cations that are commonly exchangeable. The channels are large enough to allow the passage of guest species. In the hydrated phases, dehydration occurs at temperatures mostly below about 400 °C and is largely reversible. The framework may be interrupted by (OH,F) groups; these occupy a tetrahedron apex that is not shared with adjacent tetrahedral [132]”.

Natural zeolites are found worldwide in huge commercial deposits, of volcanic and sedimentary origins. Many different types of natural zeolites have been identified around the world. Among the most common types, we found clinoptilolite, mordenite, phillipsite, chabazite, stilbite, analcime and laumontite, clinoptilolite being the most abundant and widely used [133]. Zeolites present a unique structural chemistry (e.g., Si/Al ratio, pore size), high specific surface area, cation exchange capacity and thermal stability, which enables their application in different fields such as adsorption, catalysis, building industry, agriculture, soil remediation, and energy [133,134].

#### 4.2.2. Structure and Properties

**Figure 3:**
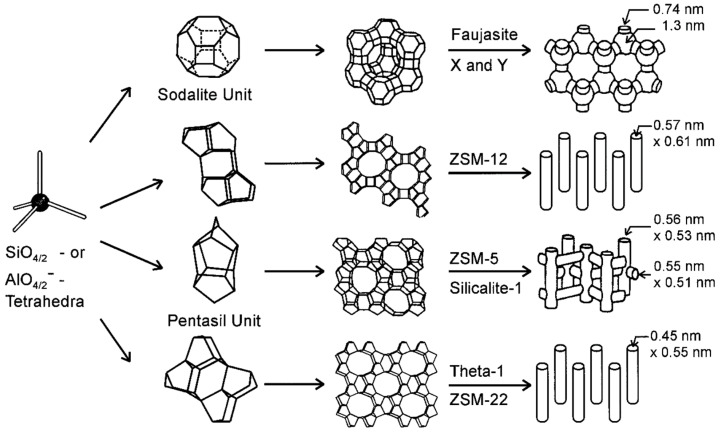
Structures of four selected zeolites (from top to bottom: faujasite or zeolites X, Y; zeolite ZSM-12; zeolite ZSM-5 or silicalite-1; zeolite Theta-1 or ZSM-22) and their micropore systems and dimensions [134] (reprinted from Publication Solid State Ion. 131, J. Weitkamp, Zeolites and catalysis, 175–188 (2000) with permission from Elsevier).

The elemental structure of alumina–silicate type zeolites consists of an aluminosilicate framework with a tetrahedral arrangement of silicon cations (Si^4+^) and aluminum cations (Al^3+^), surrounded by four oxygen anions (O^2−^). In this arrangement, each oxygen ion connects two cations and is shared between two tetrahedrons; thus, yielding a macromolecular three-dimensional framework of SiO_2_ and AlO_2_ tetrahedral building blocks [135]. The substitution of some Si^4+^ ions by Al^3+^ results in a net negative charge in the tectosilicate framework that arises from the different formal valency of (AlO_4_)^5−^ and (SiO_4_)^4−^ tetrahedrons and is normally located on one of the oxygen anions connected to an aluminum cation. Consequently, counter-ions (usually alkaline or alkaline earth metals) are found on the external surface of zeolite, bound by weak electrostatic interactions, to balance the negative charges in the structure [135]. These cations are commonly exchangeable and are situated in the cavities generated in the 3-dimensional bond tetrahedral framework, which constitutes a wide range of polyhedra structures, which in turn form the different extended frameworks of the various zeolite crystal constructions (Figure 3).

The porosity in zeolites is a product of the interlinked cages that are opened to the external surface of the particle and is normally below 2 nm; hence, zeolites are microporous materials [135]. The interstices hold not only cations but also large amounts of water and because zeolites are crystalline, the pores are as regular as the positions of the lattice atoms forming their walls [136]. Then, only molecules of the right size and shape can be copiously sorbed and that is why zeolites are termed molecular sieves [136].

Natural and modified zeolites have been used as catalysts or adsorbents due to their large surface area, crystallinity, thermal stability, defined pore size (which allows shape selectivity for particular molecules), cation exchange capacity and presence of Brönsted or Lewis acid sites. These two last characteristics will be briefly discussed:

*Cation exchange capacity: Ion exchange proceeds in an isomorphous fashion, according to the following reaction:

(6) $$z_{B}A^{z_{A}{}^{+}}+z_{A}BL_{z_{B}}↔z_{A}B^{z_{B}{}^{+}}+z_{B}AL_{z_{A}}$$

where *zA^+^* and *zB^+^* are the valences of the respective cations, and L is defined as a portion of the zeolite framework holding unit negative charge.

The extension of the ion exchange reaction depends on several factors such as ion charge and size, charge density of the anionic framework (related to the amount of Si^4+^ replaced) and concentration of the external electrolyte solution [133]. Different cations polluting water (e.g., Cu^2+^, Ag^+^, Zn^2+^, Cd^2+^, Hg^2+^, Pb^2+^, Cr^3+^, Mo^2+^, Mn^2+^, Co^2+^, Ni^2+^, NH^4+^) can be exchanged with biologically acceptable cations present in the exchange sites of zeolites, such as Na^+^, K^+^, Mg^2+^, Ca^2+^ or H^+^ [137]. However, most organic molecules are too large to penetrate into the channels and cages that conform to natural zeolites structures and are not able to access the extra-framework exchange sites [137]. Synthetic materials may be a better option in these cases.

*Acid sites in zeolites: There are different aspects defining the activity of the material, such as the chemical nature (Brønsted versus Lewis), density, strength and accessibility of the acid sites [138].

Brønsted acid sites can be located in: (i) weakly bound protons on SiOH groups at tetrahedrally coordinated silicon atoms interacting with neighboring atoms acting as Lewis acid sites (electron pair acceptors, such as aluminum atoms), (ii) silanol groups (SiOH), which are located on the external surface of crystal particles or at framework defects, (iii) hydroxyl groups formed at extra-framework aluminum species [138]. Moreover, bridging hydroxyl groups (SiOHAl) are responsible for the presence of strong Brønsted acid sites in zeolites [139].

Framework Lewis acid sites are discussed to consist of positively charged silicon ions in the neighborhood of tri-coordinated aluminum atoms. In addition, dealumination of the zeolite framework may be accompanied by the formation of Lewis acid sites at extra-framework aluminum species and framework defects [138].

Dealumination by calcination, hydrothermal treatment or drastic acid digestion is the most important reason for the formation of framework defects and silanol groups [138].

#### 4.2.3. Modification of Natural Zeolites

Natural zeolites present some limitations in comparison to the synthetic versions of the material, such as the presence of impurities and the lack of consistency or characterization study regarding the composition of the product commercialized. Therefore, this material is often modified through simple procedures to purify it, incorporate active species or to increase its acidic character or surface area:

(i) Purification: Simple acid washing can remove impurities. In addition, purification usually involves available mineral processing equipment such as jaw crushers, crushing rolls, hydrocyclones, classifiers and shaking tables [140].
(ii) Thermal Reduction: Is usually during the preparation of bifunctional catalysts. The zeolite is exchanged with the cationic form of the metal that will be used as the catalyst, and afterward is reduced in H_2_ atmosphere [140].
(iii) Acid treatment with mineral acids: The acid treatment may remove the impurities that are blocking pores in the structure, leading to an increase in the specific surface area and microporosity of the material. In addition, cations are exchanged for H^+^, obtaining a proton-exchanged zeolite. Depending on the aggressiveness of the treatment (acid strength, temperature), it can lead to a progressive dealumination of the material, which causes a reduction in the cation exchange capacity and under drastic conditions, to the collapse of the structure [133]. On the other hand, a higher Si/Al ratio increases the hydrophobic character of the material, which is interesting in applications related to the adsorption/separation of non-polar molecules [133].
(iv) Ammonium exchange: Another modification procedure to obtain a proton-exchanged zeolite is the ammonium exchange followed by calcination, leading to desorption of ammonia and the bonding of a hydroxyl proton at the bridging oxygen of an Si–O–Al arrangement [138]. Ammonium exchange can maintain the structure while acid treatment generally results in dealumination and reduction in thermal stability [133].
(v) Modification by surfactants: Cationic (organic) surfactants are used to extend the adsorption capacity of zeolites to a broader spectrum of pollutants (for example, organic molecules and anionic compounds) [141]. Surfactants form a monolayer or “hemicelle” at the solid–aqueous interface via strong ionic bonds at surfactant concentrations below the critical micelle concentration (CMC). The hydrophobic tails of the surfactant molecules associate to form a bilayer when the concentration exceeds the CMC. These different coverings are favorable for the adsorption of different compounds. It has been reported that the removal of hydrocarbons is optimal at the point of full monolayer coverage [141]. On the other hand, anionic adsorption occurs in the bilayer configuration [141].
(vi) Heat treatment (T ≥ 773 K) or steaming of acidic zeolites: This causes dihydroxylation of Brønsted acid sites and formation of Lewis acid sites. When framework dealumination occurs, the formation of Lewis acids sites can be attributed to the appearance of extra-framework aluminum species of octa-, penta-, or tetrahedral coordination [138].
(vii) Hydrothermal Treatments (zeolite synthesis): Treatment with highly basic sodium or potassium hydroxide solutions causes amorphization and change in chemical composition; hence this treatment produces a highly active aluminosilicate gel, very useful for succeeding zeolite synthesis using as raw material the amorphous solid phase [140]. Synthetic zeolites are more expensive compared to the similar natural counterparts; however, they present a higher cation exchange capacity and improved tunable acidity and pore size (for example in hierarchical zeolites).
(viii) Generation of a hierarchical structure: The presence of only microporous channels in zeolites may restrict diffusion, limit the selectivity range of desired products and promote deactivation by coking. Therefore, different synthesis or modification strategies have been studied to obtain hierarchical structured zeolites with not only their inherent microporosity, but also mesoporosity or even macroporosity [139,142]. In general, these methods can be subdivided in two main groups: (I) the top-down, where mesopores can be created in available zeolites by etching away a part of it, and in some cases recrystallizing again and (II) the bottom-up methods, where a hierarchical system is created during the zeolite synthesis [143]. From the top-down methods, dealumination is the oldest technique, which can be performed by means of steam, acid, or heat treatment [143]. However, there are some important disadvantages such as partial amorphization of the zeolite, wide mesopores size distribution and the existence of cavities and mesopores not connected to the surface. Another alternative is desilication, where the zeolite is contacted with an alkaline solution. The Si-O-Si bonds are selectively hydrolyzed causing the preferential removal of Si from the zeolite framework and the formation of mesopores. However, when using too concentrated alkaline solutions, a lot of zeolite material can be lost and microporosity could decrease drastically [143].

#### 4.2.4. Application of Natural Zeolites in Ozonation Processes

Table 2 presents the operating conditions and degradation efficiency outcomes obtained in the catalytic ozonation of different organic pollutants using natural zeolites.

Several authors have reported the use of natural zeolites mostly as an accessible, cost-effective support. For example, Gümüs and Akbal [144] reported the use of clinoptilolite as a catalyst support for the ozonation of humic acid. The zeolite was crushed and sieved, washed with HCl (1 N), impregnated with FeCl_3_ (at controlled pH with NaOH), and finally carefully washed again and dried. The iron oxide coating was composed of small particles on top of the natural zeolite at the micron scale. The surface hydroxyl groups at the water/oxide interface interacted with O_3_ generating a series of radical chain transfers. The catalyst increased the decomposition of humic acid compared to ozonation alone and it was used for at least three cycles without significant reduction in performance. Fe leaching was observed but concentrations were lower than the drinking water permissible limits of the WHO (Secondary Maximum Contaminant Level (SMCL) of 300 µg L^−1^).

Zhang et al. [145] also used a natural zeolite as a support in the synthesis of a cerium-loaded catalyst for the ozonation of penicillin G (PG). The natural material was treated with a HNO_3_ solution (1 mol/L), washed and dried. Then, the acid modified zeolite was impregnated with Ce(NO_3_)_3_ solution at controlled pH, dried and calcined. The final material was characterized by several techniques (TEM, SEM-EDS, XRD, BET and XPS). The authors observed that the catalyst retained the high mesopore framework of the natural zeolite and agglomerated cerium oxide particles were non-uniformly distributed on the acid-treated zeolite surface. According to the control oxidation experiments, the pollutant’s degradation efficiency in the catalyzed system was higher than that of O_3_ alone. Radical scavenging tests revealed that hydroxyl and superoxide radicals were the dominant reactive oxygen species in the degradation process. Five cycle stability tests were performed and a gradual loss of activity was observed, related to cerium ion leaching and adsorption of reaction intermediates on active sites.

**Table 2:** Natural zeolites as catalysts in the ozonation of organic pollutants.

Catalyst	Pollutant	Operating Conditions	Reaction Outcomes	Ref.
Iron-coated zeolite (clinoptilolite) (ICZ)	Humic acid (HA)	Semibatch reactor 2 L, gas flow rate 2 L/min, O_3_ 10 mg/L, 24 °C, 30 mg/L HA, catalyst load 0.75 g/L, 60 min, pH_0_ 6.5.	COD Conversion: Adsorption: 7.15%. O_3_ alone: 21.39%. Catalytic ozonation with IZC: 62%. Catalytic ozonation with natural zeolite: 22.31%. Fe leached 54.59µg/L	[144]
Cerium-loaded natural zeolite (CZ)	Penicillin G (PG)	Semibatch reactor 1 L, gas flow rate 0.6 L/min, O_3_ dose 6 mg/min, 25 °C, 50 mg/L PG, catalyst load 2 g/L, 120 min, pH_0_ 4.5.	TOC Conversion: Adsorption: 4%. O_3_ alone: 9%. Catalytic ozonation: 22%. Ce leached in cycles of 20 min: 0.057 mg/L (1st cycle), 0.246 mg/L (5th cycle).	[145]
Natural zeolite (Chilean mining company “Minera Formas)	O_3_ decay	Differential circular flow reactor: after reaching dissolved O_3_ saturation (125 µM, 20 °C) the gas flow (2 L/min) is closed and begins liquid recirculation (1.5 L/min, 1 L) through the fixed bed (19 mL, 15 g/L).	Apparent first order ozone decay constants (×10^3^ s^−1^): O_3_ alone: 0.5 (pH 2), 0.6 (pH 7), 3 (pH 8). Catalytic ozonation: 0.9 (pH 2), 1 (pH 7), 3.6 (pH 8).	[89]
Acid-treated natural zeolite (53% clinoptilolite, 40% mordenite, 7% quartz) (AZ)	Methylene blue	Differential circular flow reactor: after reaching dissolved O_3_ saturation (125 µM, 20 °C) the gas flow (2 L/min) is closed, MB is added (94 × 10^−6^ M) and begins liquid recirculation (1.5 L/min, 1 L) through the fixed bed (19 mL, 15 g/L), pH_0_ 6.	Pseudo first order rate constants of MB removal (×10^3^ s^−1^): O_3_ alone: 0.5, O_3_ + AZ: 5, AZ:1.9.	[146]
Montanit300^®^ (M, natural volcanic stone, rich with zeolite clinoptilolite) and acid treated Montanit300^®^ (5MH45 with HCl and 80MS with H_2_SO_4_)	Orange II (OII)	Semibatch reactor 1 L, O_3_ dose 14 mg/(L min), 24 °C, 0.5 L of 100 mg/L OII, catalyst load 1 g/L, 240 min, pH_0_ 6.	TOC Conversion: Adsorption: Negligible. O_3_ alone: 66%. Zeolite + O_3_: 91% (M), 65% (5MH45), 88% (80MS). Fe leached (mg/L): 0.95 (M), 0.5 (5MH45), 0.8 (80MS). Mn leached (mg/L): 0.066 (M), 0.0037 (5MH45), 0.0074 (80MS).	[81]
Natural zeolite Faujasite	Landfill leachate	Semibatch reactor, O_3_ dose 27 mg/m^3^, <15 °C, 0.5 L of 2500 mg/L COD, catalyst load 160 g/L, 100 min, pH_0_ 5–9.	COD Conversion: Adsorption: 66% (pH 8.2). O_3_ alone: 45% (pH 9), 30% (pH 5). Zeolite + O_3_: 65% (pH 11), 50% (pH 5). Amonia Conversion: Adsorption: 57% (pH 8.2). O_3_ alone: 28% (pH 9), 10% (pH 5). Zeolite + O_3_: 20% (pH 11), 35% (pH 5).	[147]

**Figure 4:**
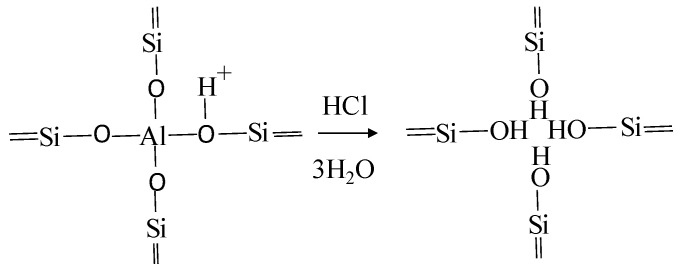
Acid treatment of zeolites.

Other authors tested natural zeolites not as a simple support, but as the catalytic material. For example, Valdés et al. [89] studied the catalytic decomposition of ozone in aqueous medium, promoted by natural zeolite and volcanic sand. The natural zeolite was treated with 2 M HCl–hydroxylamine solution. The acid treatment increased the surface area and the amount of Lewis acid sites, which was related to structural changes involving aluminum expulsion in a soluble form, and its replacement by a nest of four hydroxyl groups (Figure 4). As a result, the zeolite point of zero charge decreased from 7.9 to 2.7. It was observed that the strong Lewis acid sites could act as initiators and/or promoters of radical chain reactions, accelerating ozone decomposition.

Valdés et al. [146] also studied the effect of the surface properties of natural zeolites on the kinetics of the heterogeneous catalytic ozonation of Methylene blue (MB).

A Chilean natural zeolite was acid-treated using HCl (2.44 M) and thoroughly characterized by several techniques (N_2_ adsorption at 77 K, XRF, acidimetric–alkalimetric titration, NH_3_ and CO_2_ TPD, Fourier transform infrared spectroscopy of pyridine adsorption) to evaluate surface area, surface charge, pH_pzc_ and nature and strength of acid sites. According to the characterization results, the acid treatment increased the surface area and transformed the chemical surface composition of the natural zeolite into a surface reach in acid sites. The reduction in the aluminum content after acid treatment decreased the charge density of the anion framework. Thus, the pH_PZC_ was reduced from 8.7 to 2.9. Then, the authors explained that the hydroxyl groups on the acid treated zeolite were subjected to less intense interaction with the framework, facilitating deprotonation, i.e., enhancing the acid strength. The oxidation results were modeled using a set of two homogeneous and three heterogeneous surface reactions, analyzing the quantitative effects of single ozonation, adsorption and coupled treatments, on MB removal rate, together with the effect of pH and the presence of radical scavengers. The heterogeneous catalytic ozonation promoted by the modified natural zeolite involved specific chemical interactions between the zeolite surface, dissolved organic pollutants and the oxidants. In the pH range studied, MB molecules were positively charged. Under such conditions, when pH < pH_PZC_, adsorption of MB molecules could relate to a combined mechanism including cationic exchange, surface complexation phenomena and van der Waals forces, overcoming repulsion effects between cationic MB molecules and protonated surface hydroxyl sites. However, the surface complexation mechanism could play a fundamental role at pH > pH_PZC_. MB molecules could be adsorbed by coordination to the surface of the zeolite and then react with oxidating agents, such as molecular ozone and ozone decomposition by-products, since the active acid sites could effectively convert aqueous ozone into radicals. In the presence of the acid-treated zeolite, dissolved ozone could act as a Lewis base, being adsorbed on “true” Lewis acid sites (such as extra-framework aluminum species) on the zeolite surface. Additionally, at pH conditions higher than pH_PZC_, ozone could act as a Lewis acid, being adsorbed on deprotonated surface hydroxyl groups, enhancing radical generation. In this work, maximum MB removal rates were obtained at pH>pH_PZC_, where hydroxyl groups remained deprotonated.

Inchaurrondo et al. [81] studied the catalytic ozonation of an azo-dye, Orange (OII), using a natural aluminosilicate rich in natural zeolites: Montanit300^®^. The material was used without a previous treatment (M sample) and modified through acid digestions with HCl (5MH45 sample) or H_2_SO_4_ (80MS sample). According to the characterization results, the 5MH45 and 80MS samples presented higher specific surface area, 5MH45 showed slightly higher quantity of acid sites and 80MS showed higher Si:Al ratio, which is usually related to a stronger hydrophobic character. However, according to EDX and XRF results, these samples also showed a reduced content of Fe and Mn, elements that have been linked to catalytic activity in ozonation. The adsorption contribution was negligible for all samples. The activity of M and 80MS was associated to Mn leaching promoted by the interaction between the catalyst surface and carboxylic acids. Manganese was present in trace levels on these materials (undetected by the initial EDX measurements) and leaching concentrations of only 0.066–0.0037 mg/L were measured. These low concentrations had a huge impact on the degradation results and, therefore, the presence of impurities should be carefully assessed in materials of natural origin. The reaction mechanism was based on a dynamic cycle involving reduced and oxidized forms of Mn, which was governed by the oxidant dose and nature of the reaction intermediates. TBA decreased the mineralization levels suggesting a reaction mechanism involving radicals. For the M sample, the mineralization level was mostly sustained over the four runs studied. Moreover, a fraction of the leached Mn may have been recovered as precipitated MnO_2_, throughout stability cycles. At neutral pH, M showed no activity and 80MS showed moderate activity, which could be associated to the enhanced interaction between the catalyst surface and ozone/pollutants, due to its higher Si:Al ratio (higher hydrophobicity).

AlGburi et al. [147] employed a natural zeolite (Faujasite) as a catalyst in the ozonation of landfill leachate. The authors studied the effect of pH and observed that at lower values Faujasite acted as a reactive surface that adsorbs pollutants and ozone, promoting their reaction. For COD and color, higher removal was achieved under alkaline conditions. According to the authors, at basic pH the surface of zeolite enhances ozone decomposition and the formation of hydroxyl radical.

### 4.3. Oxides

#### 4.3.1. Structure and Properties

Among natural oxides typically present in soils, there are oxides, hydroxides, oxyhydroxides, and hydrated oxides of Si, Fe, Mn, Al, and Ti [148]. The basic structural unit of Fe, Mn, Al and Ti oxides are cationic metal centers bound to six oxygens in octahedral configuration. The octahedra may be linked to each other in three ways, sharing oxygen corners (1 O), sharing edges (2 O) or faces (3 O). In the case of silicon, the element is bound to four oxygens, forming tetrahedra, which are connected via corners only [148].

**Figure 5:**
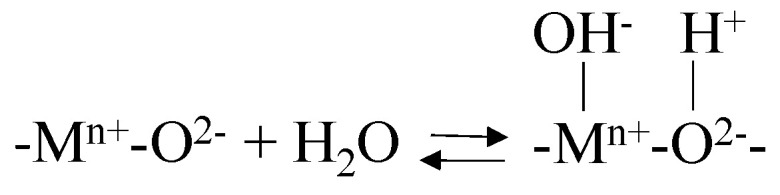
Interaction between a water molecule and the surface coordinatively unsaturated metal cations and oxygen anions.

The catalytic properties of oxides are mainly determined by their acidity or basicity character. This is often analyzed in terms of Brönsted or Lewis acid/base sites. Brönsted acidity–basicity is defined as the ability of proton abstraction–acceptation and Lewis acidity–basicity is the ability of electron acceptation–abstraction [149]. These sites can be found in the partially hydroxylated oxide surface, which is characterized by the presence of hydroxyl groups and coordinatively unsaturated metal cations and oxygen anions. These exposed coordinatively unsaturated metal cations and oxygen anions on the surface of oxides are Lewis acid and Brönsted conjugate base sites, respectively [150]. Brönsted acid sites are associated with the presence of hydroxyl groups. Therefore, the number of such sites depends on the extent of hydroxylation of the surface, which involves the interaction between a water molecule and the surface coordinatively unsaturated metal cations and oxygen anions (Figure 5) [150].

Since surface coordinatively unsaturated ions are important, it follows that the presence of cation or anion vacancies and other defects that result in greater exposure of the ions also affects acidity [150]. Therefore, the nature of the cation (size ad charge), crystallinity, and presence of impurities or surface defects, determine the reactivity of the material. For example, oxides of Fe, Mn and Al may exhibit a high surface area with reactive surface sites, while sand-sized crystals of Si oxide quartz are chemically very inert [148].

The majority of the studies addressing the use of oxides as catalysts in the ozonation process are based on synthetic Al, Fe and Mn materials and the use of natural oxides is scarce. Therefore, the catalytic behavior of synthetic oxides will be presented together with studies based on natural oxides, to deeply understand the full potential of these materials.

The outcomes obtained with oxides of natural origin are presented in Table 3.

#### 4.3.2. Aluminum Oxides

Alumina is one of the most studied oxides in the ozonation process. Aluminum oxides have high surface areas (up to 600 m^2^/g), high pH_PZC_ (pH 8–10), and a pH-dependent surface charge [148]. Alumina presents a high adsorption capacity (heavy metals and anions) related to the reactive surface area (singly coordinated OH groups at crystallite edges) rather than the total surface area [148].

There are numerous studies focused on unraveling the catalytic role of alumina in the ozonation process, mostly based on synthetic materials [45,47,78,151]. Then, a brief description of relevant studies regarding the catalytic ozonation mechanism using synthetic aluminum oxides, will be presented. Among these studies, a great number have proposed a mechanism based on the generation of radical species from the interaction between ozone and surface hydroxyl sites. An example of this mechanism is presented in the study performed by Ernst et al. [45], focused on the catalytic ozonation, in buffered and non-buffered solutions, of refractory organic compounds: oxalic, acetic, salicylic and succinic acids, using aluminum oxide. According to the results obtained, the authors proposed a mechanism based on the adsorption of ozone on the catalyst surface and its decomposition in the presence of hydroxyl sites. This study described that O_3_ decomposition may generate active atomic oxygen, which reacts with surface hydroxyl groups to produce O_2_H^−^ anions. Then, the anions can react rapidly with another O_3_, generating O_2_H radicals or, also, the O_2_H radicals could be produced directly. This radical can react subsequently with another ozone molecule to generate O_3_^−^. The ozonide radical decomposes into oxygen and a free OH radical, which can oxidize organic compounds either in solution, or on the surface or in a thin film layer above the catalyst’s surface. In this case, the adsorption of organic compounds is not necessary, on the contrary, would be detrimental, since there would be an overlaying of hydroxyl groups. The authors observed that the catalytic effect was more pronounced for succinic acid, since it showed a low adsorption affinity in both buffered and non-buffered experiments. Then, from this study, it is important to highlight that the nature of the organic compound could define the importance of the catalytic effect versus adsorption and/or single ozonation.

Qi et al. [78] also proposed a radical based mechanism to explain the catalyzed ozonation of 2-methylisoborneol (MIB) by different aluminum oxides (γ-AlOOH and γ-Al_2_O_3_). The surface hydroxyl groups were also the designated active sites, showing higher activity at the point of zero charge. Radical scavenger experiment results indicated that the catalyzed ozonation by γ-Al_2_O_3_ followed a hydroxyl radical (HO•) reaction pathway whereas γ-AlOOH followed a solid surface mechanism. However, the authors performed O_3_ decomposition tests, without MIB, and observed that both γ-AlOOH and γ-Al_2_O_3_, in this case, could enhance ozone decomposition to generate hydroxyl radicals. This was explained considering that MIB interacted with surface hydroxyl groups and that the adsorption capability of γ-AlOOH was higher than that of γ-Al_2_O_3_. Therefore, in the case of γ-AlOOH, the participation of surface hydroxyl groups in MIB adsorption restrained its capability to catalyze ozone decomposition to generate HO•. Then, from this study, it is interesting to highlight that materials with similar composition but different surface texture and chemical properties could show quite different reaction mechanisms.

Another interesting and detailed study was presented by Vittenet et al. [47] on the role of mesoporous alumina in the ozonation of 2,4-dimethylphenol (2,4-DMP) (petrochemical refractory molecule). In tests performed with and without γ-Al_2_O_3_, the promotion of HO• radicals due to basic pH was discarded, due to the very low OH^−^ concentration at the studied pH. This was checked in tests performed in the presence of a radical scavenger, TBA, which did not inhibit TOC removal in single ozonation tests. On the other hand, in the presence of alumina, t-BuOH induced a decrease in TOC conversion. This indicated that γ-Al_2_O_3_ promoted the formation of HO• as no t-BuOH was adsorbed on the material. TOC removal with γ-Al_2_O_3_ was very fast during the first hour of ozonation, much faster than single ozonation, and then stopped due to the inhibition of all Al-OH groups by carboxylates. As the reaction continued, a similar evolution to single ozonation was obtained, which allowed the authors to propose that only Al-OH basic sites were involved in the catalytic mechanism.

Stability tests showed a decay in TOC removal with a remaining stable activity, due to either reversible catalytic activity of Lewis sites or to a higher efficiency of direct O_3_ reaction at the solid surface. This last statement can be related to a reaction mechanism proposed by several authors, centered on the complexation of organic pollutants on the alumina’s surface, rendering more reactive compounds easily oxidized by ozone molecules [42]. For example, Kasprzyk-Hordern et al. [54] studied the ozonation of natural organic matter (NOM) on alumina and proposed a mechanism based on a dynamic balance between continuous adsorption and oxidation of NOM by O_3_ at the solid–liquid interface. This hypothesis was reinforced by the high NOM concentrations employed and the high affinity of these compounds towards alumina that might block, through adsorption, active surface sites and as a result make them inaccessible for ozone. In addition, it is also possible that NOM adsorption generated a hydrophobic layer on the surface of alumina, promoting its interaction with ozone. Furthermore, Pocostales et al. [31] studied the catalytic ozonation of different pharmaceutical compounds in the presence of a commercial γ-Al_2_O_3_ and a synthesized Co_3_O_4_/Al_2_O_3_ catalyst. The catalytic behavior of the materials was attributed to two pathways: ozone chemisorption and decomposition into free radicals and adsorption of some organic compounds that, once adsorbed, could react more easily with ozone.

In some studies, the alumina’s adsorption capacity would hinder the catalytic activity, as reported by di Luca et al. [152]. Even though the material could increase O_3_ decomposition in the absence of organics, the enhanced TOC removal during the ozonation of the antibiotic sulfamethoxazole was mostly attributed to the adsorption of the reaction by-products, often carboxylic acids. In this case, single ozonation was employed to increase the molecular polarity of oxidized compounds, which were more feasibly adsorbed onto the alumina surface.

According to this collection of studies, it can be concluded that the catalytic role of alumina depends on the compound and particular operating conditions chosen, such as ozone dose and pH.

Regarding aluminum oxides of natural origin, only few studies are found in the literature. Among them, the studies of Qi et al. [46,153] and Chen et al. [76] using raw and modified bauxite (mineral mostly composed of alumina) will be described below.

Qi et al. [46] employed raw bauxite as a catalyst for the ozonation of 2,4,6-trichloroanisole (TCA). X-ray powder diffraction (XRD) characterization showed a composition based on boehmite (major component), kaolinite and quartz. In order to study the oxidation mechanism, two types of experiments were performed: ozonation in the presence of a radical scavenger (TBA) and calculation of the ratio of HO• exposure to ozone exposure, using pCBA as a probe compound. Both experiments confirmed that HO• accounted for the enhancement of TCA degradation. The generation of HO• was inhibited faintly by the presence of natural organic matters (NOMs) and alkalinity in natural water. The concentration of aluminum leached was negligible. The presence of silicates in the composition of the natural material helped to keep aluminum complexed and not as labile.

Qi et al. [153] studied the catalytic ozonation of 2,4,6-trichloroanisole (TCA) by raw and metal oxide (Fe or Mn) modified bauxite, prepared by the incipient wetness impregnation method. Adsorption levels of TCA were all <10.0%. Then, the oxidation reaction played an important role in the pollutant’s removal. The authors observed a two-stage reaction, which occurred in both the sole ozonation and catalytic ozonation processes. The initial stage, called instantaneous ozone demand, was characterized by a fast ozone consumption, connected to the oxidation reaction between ozone (or HO•) and TCA. The second stage was a slow oxidation process where the residual molecular ozone continued to oxidize TCA or reacted with the catalyst surface, generating active oxygen species. Both stages fit a pseudo first order kinetic model and the authors studied the connection between the volume of micropore and mesopore of raw and modified bauxite, and the reaction kinetic constants from the catalyzed ozonation processes. A good linear relationship between the micropore volume and the kinetic constant of the initial reaction and a good linear relationship between the mesoporous volume and the kinetic constant of the second stage reaction were observed. A good linear dependence between the peak area of surface hydroxyl groups in FT-IR (1637 cm^−1^) and the reaction kinetic constant of the second stage reaction was also observed. From these results, the following mechanism was proposed: “First, ozone molecules and TCA were adsorbed quickly in the micropores of the catalyst in the initial reaction, and a surface catalytic reaction resulted in ozone decomposition and TCA degradation. The microporous surface increased the probability of collisions among the catalyst, pollutants, and ozone molecules. The direct and indirect oxidation between ozone and TCA occurred. After that, ozone and TCA diffused over the mesoporous surface of the catalyst. Mesoporous surface was favored for the reaction between ozone and TCA in the second reaction phase.” In addition, the enhancement of the catalytic activity with the modified bauxite in the second phase of the reaction was connected to the higher density of hydroxyl groups in the mesoporous surface (FT-IR measurements). Regarding the materials’ stability, the dissolution of Fe^3+^ was below the drinking water limit in China (0.3 mg/L) and the contribution of the homogeneous catalytic ozonation by Fe^3+^ was considered insignificant. However, in the case of the manganese-modified material, the homogeneous contribution of the cation leached was considered important.

Chen et al. [76] used raw (BO) and calcined bauxite (CBO) ores in the catalytic ozonation of p-nitrophenol. The BO was washed, dried and ground. The CBO sample was obtained after calcination at 1075 K for 6 h. The chemical compositions (XRF) of RBO and CBO showed Al_2_O_3_, SiO_2_, Fe_2_O_3_ and TiO_2_ as dominant constituents. The surface area of CBO and RBO was very small, 0.873 and 1.143 m^2^/g, respectively. The samples presented a pH_PZC_ of 7.2 in the case of RBO and 11.48, for CBO. The calcined bauxite showed more activity than the raw one, which was related to pore and surface structural changes after calcination.

The highest mineralization degree was obtained at initial pH 11.98, a value close to the pH_PZC_ of CBO. As observed in other studies, the neutral surface OH groups facilitate ozone decomposition and HO• generation. Experiments in the presence of different scavengers (phosphate, carbonate, and bicarbonate and TBA) were performed. The presence of phosphate, carbonate and bicarbonate reduced the activity due to the affinity of these compounds towards Lewis acid sites. On the other hand, the presence of TBA did not affect the reaction. From this, the authors suggested that the catalytic ozonation of p-nitrophenol primarily followed the HO• mechanism on the catalyst surface rather than in bulk solution. The COB sample showed higher stability than the raw one and proved to be active during 10 sequential runs.

#### 4.3.3. Iron Oxides

Iron oxides have been widely used as catalysts and adsorbents in processes related to water remediation [20,59,154]. In nature, iron is released by weathering of Fe(II)-containing silicates (biotite, pyroxene, amphibole, olivine) and after oxidation and hydrolysis, most of it precipitates as Fe(III) oxides [148].

Fe-based catalysts have received increasing attention due to (1) abundance in nature, (2) simple synthesis, (3) almost no toxicity, and (4) some oxides show special characteristics, such as the magnetic properties of Fe_3_O_4_ or the high density of hydroxyl sites in FeOOH [59]. Their different characteristics are determined by their composition, valence and, therefore, crystal structure. As previously pointed out, both Brönsted and Lewis acid sites have been described as the central active sites [59].

Among iron oxides existing in nature, goethite (α-FeOOH) is the ubiquitous Fe oxide mineral in soils [148]. This is attributed to its high stability, which enables its use in numerous catalytic ozonation studies [64,155]. However, the majority of the research around this oxide was performed using synthetic materials. Then, a brief discussion of the catalytic behavior of synthetic iron oxides will be presented below.

Zhang et al. [155] studied the catalytic ozonation of nitrobenzene using a synthetic goethite and investigated the relationship between the characteristics of the surface hydroxyl groups present in the hydroxylated synthetic FeOOH and their catalytic activity in promoting hydroxyl radical generation from aqueous ozone. Nitrobenzene was selected as an ozone-resistant probe since it is hardly adsorbed on the hydroxylated surfaces of metal oxides. The authors described a mechanism based on the generation of hydroxyl radicals, with the hydroxyl groups as the active centers. This was confirmed through the inhibition observed in the presence of phosphate and sulfate ions, which substituted surface hydroxyl groups (ATR-FTIR analysis). The authors compared different synthetic oxo-hydroxides such as β-FeOOH, γ-FeOOH, γ-AlOOH and α-FeOOH and observed that their catalytic activity followed the increasing order of γ-AlOOH < γ-FeOOH < β-FeOOH <α- FeOOH. No correlation could be established between the surface hydroxyl densities or other surface characteristics (surface area, pore volume) and the activity. Then, surface hydroxyl groups were further examined with the ATR-FTIR technique in D_2_O suspensions and observed that surface MeO–D bonds followed the increasing order of α-FeOOH < β-FeOOH < γ-FeOOH < γ-AlOOH, which correlated with the reversed order of activity. Then, the authors concluded that relatively weak surface FeO–H bonds lead to a higher affinity of electrophilic H and nucleophilic O towards molecular ozone, which makes it easier for surface OH–ozone combination. This was specially promoted at neutral pH (since protonation weakens O nucleophilicity and at high pH the electrophilic H is released).

Sui et al. [64] also studied the activity of FeOOH and observed the generation of hydroxyl radicals (ESR spin-trap technique) from hydroxyl sites as active centers, under acidic and neutral pH. The authors also observed the ligand exchange of hydroxyl groups by phosphate adsorption, but anions were desorbed during the catalytic ozonation process, resulting in the reactivation of activity.

Larouk et al. [156] studied the synergistic effect of the hematite-SBA-16 combination in the catalytic ozonation of Orange G: (i) hematite provided a high catalytic activity despite its low porosity; (ii) SBA-16 provided a high specific surface area for adsorption and surface reaction notwithstanding its low intrinsic catalytic activity. Hematite showed activity at low pH values; however, it was attributed to the partial dilution of Fe^3+^, which was supposed to generate Fe^2+^-alkylate complexes with high reactivity towards ozone under acidic conditions.

Yan et al. [62] presented a study comparing O_3_ transformations on different iron oxides suspensions (α-Fe_2_O_3_, α-FeOOH, Fe_3_O_4_) using FTIR of adsorbed pyridine, ATR-FTIR and electron paramagnetic resonance (EPR) spectra with isotope 18O_3_. The characterization studies indicated that ozone was electrostatically adsorbed stably at isolated hydroxyl and hydrogen-bonded hydroxyl on α-Fe_2_O_3_ and did not react with surface Fe^3+^ ions due to the blocking by these hydroxyl groups. On the other hand, different mechanisms were observed for α-FeOOH and Fe_3_O_4_, where O_3_ was adsorbed on surface Lewis acid sites competing with water and directly interacted with surface Fe^3+^ ions, to mainly convert into O_2_•^−^ and HO• due to Fe^2+^/Fe^3+^ electronic circulation.

According to the studies described above, different iron oxides have different crystal forms and thus different hydroxyl groups, which confers different reactivity in the ozonation reaction.

Regarding the studies carried out with natural iron oxides, there are two interesting research works based on the modification of magnetite (Fe_3_O_4_), species that consists of one ferrous ion and two ferric ions in its inverse spinel crystal structure [59]. This material presents not only catalytic activity, but it can also be easily separated to be reused, due to its magnetic character.

Moussavi et al. [157] tested raw and calcined magnetite as catalysts for the ozonation of a reactive azo dye: Reactive Red-120. The magnetite ore was obtained from a local mine and was only washed and calcined (700 °C for 2 h). Fe_2_O_3_, Fe_3_O_4_, SiO_2_ and CaO were the predominant constituents (85–90%) with minor contribution of Al_2_O_3_, MgO, Na_2_O and K_2_O (about 5%). Calcination led to the destruction of pyrite and the transformation of most of the magnetite (Fe_3_O_4_) into hematite (Fe_2_O_3_). In addition, it increased the BET surface area and pore volume (burned off and/or break down of some of the materials in the natural ore at high temperature). Another important modification was the increase in the pH_PZC_ from 7.6 (raw) to 11.8 (calcined), which was related to the conversion of calcium carbonate into calcium oxide. Oxidation via radical species on the surface of the catalyst was the degradation mechanism proposed (under optimum pH 11). The greater efficacy of the calcined sample was partly attributed to the composition of the material, mainly Fe_2_O_3_, in contrast to the raw material, which was composed of Fe_3_O_4_. Moreover, the basic character of the calcined catalyst surface (pHpzc = 11.8) maintained the solution pH in higher values and provided a higher density of hydroxide ions available on the surface of the catalyst. Since the hydroxide ions act as initiators of ozone decomposition, a greater amount of HO• was generated. The calcined sample preserved its catalytic properties after reuse 10 times.

Taseidifar et al. [158] also used a cheap natural magnetite, in this case modified with oxygen plasma, owing to its cleaning effect by chemical etching (higher availability of hydroxyl groups) and with argon plasma due to its sputtering effect resulting in more surface roughness (higher surface area). The plasma-treated magnetites performed better than the raw ones, in the catalytic ozonation of Basic Blue. SEM images demonstrated the formation of nano-structured magnetite on the catalyst surface after the plasma treatment. The optimum sample was effectively used in repeated runs, and it was easily recovered by an external magnetic field, with negligible Fe leaching.

Pelalak et al. [159] used natural goethite modified by non-thermal glow discharge plasma, using different gases (N_2_ or Ar). The performance of the modified goethites was evaluated in the catalytic ozonation of the sulfasalazine antibiotic (SSZ). The materials were characterized by FESEM, EDX, TEM, XRD, XPS, BET surface area, FTIR and pH_PZC_, showing an increase in the surface area (from 29.65 to 77.31 m^2^/g) and density of surface hydroxyl groups, on the treated materials. The pH_PZC_ was only slightly modified, from 7.2 to 6.9. The plasma treatment also raised the percentage of Fe atoms on the surface of the catalysts, due to the reduction in carbon impurities and alteration of the morphological structure. Moreover, FTIR analysis showed an increment in the vibrations related to Fe-OH bonds, which was also corroborated by XPS and EDX characterization. All these confirmed that the modified samples present a higher density of active hydroxyl sites (-OH), which can enhance the performance of the catalytic process. The catalysts increased the pollutant’s degradation; especially the sample treated with N_2_, that showed enhanced surface characteristics. The investigation of the reaction mechanism, using TBA and chloroform as radical scavengers, indicated the intervention of different reactive oxygen species, such as hydroxyl radicals (•OH), superoxide radicals (•O_2_^−^), and direct ozone molecules. Greater activity was observed at neutral pH, due to the higher activity of un-dissociated -OH groups. Additionally, higher pH values promoted the electrostatic repulsion between the negatively charged surface and anionic pollutants. The treated samples were more stable (reduced Fe release), because of the consolidation of ions inside the goethite molecular structure. The stability and reusability tests showed a negligible reduction in the performance of the N_2_ treated sample over four cycles (~6%).

Park et al. [160] studied the catalytic ozonation of para-chlorobenzoic acid (pCBA), chosen as a probe compound for hydroxyl radical measurements, using goethite as a catalyst. The ozone decomposition rate changed with pH, which was attributed to the different reactivity of ozone towards the three different types of surface functional groups (-FeOH_2_^+^, -FeOH, -FeO^−^), which are pH-dependent. Ozone showed higher reactivity towards a charged surface (-FeOH_2_^+^ and -FeO^−^), due to its resonance structure, which gives ozone both an electrophilic and nucleophilic character. According to the authors, the pCBA decomposition seemed to occur at three sites: (i) on the surface of the catalyst, (ii) in the catalyst–solution interface, and (iii) in the bulk solution, involving radical reactions. Along with the experimental results, pCBA decomposition occurs only on the surface of the catalyst at pH < 3 (not affected by TBA) and the hydroxyl radicals generated by the reaction between the catalyst surface and ozone were found to be the dominant oxidizing species. However, at pH > 3 the main reactions were found to take place in the catalyst–solution interface and bulk solution.

Heidari et al. [109] studied the use of limonite (iron ore, mixture of hydrated iron(III) oxide-hydroxides) in the catalytic ozonation of sulfasalazine antibiotic (SSZ). The authors improved the raw material through the plasma technique, generating nanostructures that reduced the mass transfer problems. The natural limonite (NL) was crushed, sieved and washed, to be finally treated with a non-thermal glow discharge plasma through oxygen and mixed gases (argon and oxygen) atmosphere (PTL/O_2_ and PTL/O_2_/Ar samples). The characterization of the samples demonstrated enhanced surface area, morphology, density of active surface sites, and physical stability after the plasma treatment. FESEM analysis confirmed the production of nanosized structures with uniform size and morphology owing to the cleaning and sputtering effect of O_2_ and Ar gases. The modified catalyst improved (36%) the degradation/mineralization of SSZ, compared to sole ozonation and the combination of O_3_ with H_2_O_2_. The stability results revealed a slight decrease in the catalyst efficiency from 98.8 to 91.4% after five cycles. This was related to the nanocatalyst poisoning by the pollutant or reaction intermediates. Fe leaching was reduced from 0.221 mg/L for NL to 0.062 mg/L for PTL/O_2_/Ar, due to plasma treatment. The authors studied the effect of organic and inorganic salts to confirm that reactive oxygen species, mainly hydroxyl radicals, were responsible for SSZ degradation in the catalyzed system. The evaluation of *E_EO_* (Electrical Energy per Order) showed that the catalyzed process using the PTL/O_2_/Ar sample, was the most efficient process from the viewpoint of energy consumption.

#### 4.3.4. Manganese Oxides

In nature, Mn is released by the weathering of Mn(II)-containing silicates (biotite, pyroxene, amphibole) and after oxidation of soluble Mn^2+^ to Mn^3+^ and Mn^4+^ (brownish-black Mn oxides of low solubility) [148]. Most naturally occurring Mn oxides are initially formed through microbially mediated pathways [161] and show an oxidizing potential stronger than that of O_2_, being able to oxidize different organic molecules [148]. Therefore, the use of Mn oxides in water treatment or water remediation has been extensively studied [148,161,162]. However, the oxidation rates generally decrease with increasing pH due to: (i) changes in speciation of both the organic reductant and the oxide surface, and (ii) the electron transfer is facilitated at lower pH values (protons are required for MnO_2_ reduction) [161]. Moreover, Mn oxides are only capable of oxidizing select contaminants and current reactors need to decrease by a factor of 10 in order to be competitive with more established ozone-based advanced oxidation processes [161].

MnO_2_ has proved to be very effective in ozone decomposition in the gas phase [163] and the performance of this oxide in the catalytic ozonation of pollutants in aqueous solution has attracted much attention. Very interesting studies focused on the performance of synthetic manganese oxides. The use of synthetic materials enables a clearer understanding of the effect of the oxide phase on the activity of the material, such as in the studies of Nawaz et al. [48] and Tong et al. [164].

Nawaz et al. [48] presented a study comparing six phases of MnO_2_ (α-, β-, δ-, λ-, γ- and ε-) in the catalytic ozonation of 4-nitrophenol (4-NP), at neutral pH. The activity of α-, β-, δ-, λ-, γ- and ε-MnO_2_ catalysts was similar in terms of 4-NP conversion but different in terms of mineralization: α-MnO_2_ (82.4%) > δ-MnO_2_ (73.5%) > γ-MnO_2_ (64.2%) > λ-MnO_2_ (61.8%) > ε-MnO_2_ (60.1%) > β-MnO_2_ (50.1%). A high surface area had a positive effect on MnO_2_ activity but was not the dominant factor. The point of zero charge (pH_PZC_) of MnO_2_ mainly influenced 4-NP adsorption and most of the pH_PZC_ values were lower than the solution pH (7.0), except for β-MnO_2_. The authors determined the Average Oxidation State (AOS) as an indicator of the real state of Mn in MnO_2_. This is because the real states of Mn are generally lower than +4 due to the imperfect preparation methods. Lower AOS means higher rates of Mn^3+^/Mn^4+^ in MnO_2_ materials. Accordingly, MnO_2_ with low AOS showed stronger oxidation/reduction peaks in cyclic voltammetry characterization, which benefited catalytic decomposition of ozone to generate active species. The reaction mechanism was studied through quenching experiments using different scavenging compounds: TBA, p-benzoquinones (p-BQ) and sodium azide (NaN_3_) as scavengers for HO•, O_2_^−^ and ^1^O_2_, respectively. O_2_^−^ was the main reactive species, with 60.2% contribution, then ^1^O_2_ (27.7%) and negligible contribution of HO•. Direct oxidation of O_3_ and surface oxidation may also contribute to 4-NP removal. The leaching of Mn ion (0.5–5.3 mg/L) during ozonation was also monitored, but its catalytic contribution resulted negligible. The activity decreased by 12.0% after four cycles, possible due to Mn leaching.

Tong et al. [164] investigated the catalytic ozonation of sulfosalicylic acid (SSal) and propionic acid (PPA) using different types of commercial MnO_2_ (β-MnO_2_, γ-MnO_2_ and MnSO_4_). In this case, the activity mostly depended on the nature of the organic pollutant and pH, but not quite on the type of MnO_2_. The oxides proved to be active in the ozonation of SSal at pH 1 (not at pH 6.8 or 8.5) and no activity was observed in the case of PPA. The activity of the MnO_2_ formed in situ (using MnSO_4_) was slightly higher than that of β-MnO_2_ and γ-MnO_2_, probably related to: (1) larger surface area; (2) in situ formed MnO_2_ can function as a coagulant.

The catalytic effect of leached Mn was not important in this system. The authors observed that there was no direct relationship between the activity of the metal oxides in ozone decomposition and the activity related to the catalytic ozonation of organic compounds.

Another interesting study using a commercial oxide was presented by Andreozzi et al. [51] centered on the catalytic ozonation of oxalic acid at pH 3.2–7. The authors proposed a reaction mechanism based on the formation of a surface complex with the oxalic molecule, under moderately acidic pH, followed by a ‘one electron’ exchange step and by the detachment of the reduced surface metal center. The adsorbed or dissolved ozone could react with the surface complex -Mn^III^C_2_O_4_^−^ at a rate at least comparable with that of the intramolecular electron transfer. Additionally, O_3_ played an important role in re-oxidizing the reduced Mn species.

Again, as observed with Al and Fe oxides, activity varies with the oxide phase and the chosen pollutant and operating conditions.

Ozonation studies using natural Mn ores were presented by Chen et al. [165] and Van and Trinh [166]. In addition, Luo et al. [167] employed a Mn-modified silicate ore as a catalyst in the ozonation reaction. The main results are presented below.

**Table 3:** Natural oxides as catalysts in the ozonation of organic pollutants.

Catalyst	Pollutant	Operating Conditions	Reaction Outcomes	Ref.
Fe and Mn modified bauxite (IMB and MMB)	2,4,6-Trichloroanisole (TCA)	Batch reactor 1 L, O_3_ dissolved concentration 0.62 mg/L, room T, 28.2 µg/L TCA, catalyst load 0.5 g/L, 60 min, pH 6.5.	TCA Conversion: O_3_ alone: 56%, Bauxite + O_3_: 72.4%, MMB + O_3_: 85.5%, IMB+O_3_: 99%. Adsorption < 10%. Leaching: Mn 0.065 mg/L, Fe 0.02 mg/L (30 min).	[153]
Raw bauxite	2,4,6-Trichloroanisole (TCA)	Semibatch reactor 3 L, O_3_ dissolved concentration 0.5 mg/L, 20 °C, 100 ng/L TCA, catalyst load 0.2 g/L, 10 min, pH 6.	TCA Conversion: O_3_ alone: 34.6%, Bauxite + O_3_: 86%, γ-AlOOH+O_3_: 77%, γ-Al_2_O_3_ + O_3_: 60%. Adsorption 10%. Al leaching < 0.05 mg/L.	[46]
Raw (RBO) and calcined (CBO) bauxite ores	p-Nitrophenol (NP)	Semibatch reactor, O_3_ 2.46 mg/min, 0.1 L of 300 mg/L NP, catalyst load 5 g/L, 10 min, pH_0_ 5.	COD Conversion: O_3_ alone: 48%, RBO + O_3_: 58%, COB + O_3_: 73.5 Adsorption negligible.	[76]
Magnetite ore	Reactive Red-120 (RR-120)	Semibatch reactor, O_3_ 1 mg/min, 25 °C, 0.1 L of 100 mg/L RR-120, catalyst load 0.2 g, 10 min, pH 11.	RR-120 Conversion: O_3_ alone: 40%, raw magnetite + O_3_: 66%, calcined magnetite + O_3_: 84.5%. Adsorption 13%.	[157]
Natural magnetite modified by oxygen and argon glow discharge plasma	Oxazine dye Basic Blue 3 (BB3)	Batch reactor, O_3_ dissolved concentration 1.2 mg/L, 0.25 L of 90 mg/L BB3, catalyst load 0.6 g/L, 15 min, pH_0_ 6.7.	BB3 Conversion: O_3_ alone: 51.02%, raw magnetite + O_3_: 63.78%, modified magnetite + O_3_: 93.47%. Adsorption < 7%. Fe leaching 0.2 mg/L (O_3_ 0.3 mg/L and 20 min reaction).	[158]
Plasma-treated goethite nanoparticles: natural (NG), using N_2_ (PTG-N_2_) and using Ar (PTG-Ar)	Sulfasalazine antibiotic (SSZ)	Semibatch reactor, gas flow rate 1 L/h, O_3_ 5 mg/L in gas phase, 25 °C, 10 mg/L SSZ, catalyst load 1.5 g/L, 40 min, pH 7.	SSZ Conversion: O_3_ alone: 61.44%, O_3_ + NG: 75.64%, O_3_ + PTG-Ar: 93.47%, O_3_ +PTG-N2: 96.05%. TOC Conversion: O_3_ alone: 31.54%, O_3_ + NG: 38.41%, O_3_ + PTG-Ar: 52.32%, O_3_ + PTG-N_2_: 56.69%. Adsorption < 10%. Fe leached: 0.18 mg/L (NG), 0.079 mg/L (PTG-N_2_).	[159]
Goethite	Para-chlorobenzoic acid (pCBA)	Batch reactor, O_3_ dissolved concentration 3 mg/L, p-CBA 1.2 mg/L at pH 3 and 3 mg/L at pH 2, catalyst load 5 g/L.	p-CBA Conversion (pH 3, 1 min): O_3_ alone: 70%, goethite + O_3_: 86%. p-CBA Conversion (pH 2, 10 min): O_3_ alone < 1%, goethite + O_3_: 16%.	[160]
Limonite: raw (NL) and modified through plasma O_2_/Ar (PTL/O_2_/Ar)	Sulfasalazine antibiotic	Semibatch reactor, gas flow rate 1 L/h, O_3_ 15 mg/L in gas phase, 0.1 mM SSZ, catalyst load 1.5 g/L, 50–120 min, pH_0_ 7.	SSZ Conversion (50 min): O_3_ alone: 62.8%, O_3_ + NL: 74.9%, O_3_ + PTL/O_2_/Ar: 98.8%. TOC Conversion (120 min): O_3_ alone: 42.5%, O_3_ + NL: 54.1%, O_3_ + PTL/O_2_/Ar: 78.5%. Adsorption 7.4–10.6%. Fe leached (50 min): 0.221 mg/L (NL), 0.062 mg/L (PTL/O_2_/Ar).	[109]
Manganese sand ore	Aniline	Semibatch reactor, O_3_ 1.76 mg/min, 25 °C, 0.1 L of 200 mg/L aniline, catalyst load 3 g/L, 10 min, pH_0_ 7.2.	COD Conversion: O_3_ alone: 42.6%, raw manganese ore + O_3_: 61%, calcined manganese ore + O_3_: 67.6%. Adsorption < 10%.	[165]
Manganese ore	Landfill leachate	Semibatch column reactor, air flow rate 7 L/min, O_3_ 2.882 g/h, 1 L of 3083 mg/L COD landfill leachate, catalyst load 0.6 g/L, 100 min, pH_0_ 8.	COD Conversion: O_3_ alone: 41.37%, manganese ore + O_3_: 61%.	[166]
Silicate ore (SO) and manganese-modified silicate ore (MnSO)	Ciprofloxacin (CIP)	Semibatch reactor 0.5 L, gas flow rate 0.3 L/min, O_3_ 0.4 mg/min, 20 °C, 0.4 L of 20 mg/L CIP, catalyst load 0.5 g/L, 30 min, pH_0_ 7.	TOC Conversion: O_3_ alone: 28%, SO + O_3_: 40%, MnSO + O_3_: 49%, MnSO + O_2_: 10%, SO + O_2_: 15%. Mn leaching 0.069–0.097 mg/L.	[167]

Chen et al. [165] employed a manganese sand ore as a heterogeneous catalyst for the ozonation of organic contaminants in petrochemical wastewater. The material was washed, ground and calcined (1023 K, 4 h). The calcination increased the surface area, pore volume and average pore size of the sample. The dominant chemical constituents were MnO (18–20%), Fe_2_O_3_ (10–12%), SiO_2_ (32–49%) and Al_2_O_3_ (3–4%). The higher SiO_2_ content in the calcined sample suggested that calcination had a stabilizing effect. The raw and calcined materials were initially tested in the ozonation of an aniline as a model compound. The calcined material showed the best degradation results and this was connected to the pore structure changes and increased surface area. Aniline degradation markedly decreased in the presence of different radical scavenging compounds: TBA, bicarbonate, phosphate and carbonate, which indicated a mechanism dominated by the generation of hydroxyl radicals. Phosphate, carbonate and bicarbonate show high affinity towards Lewis acid sites, so prevented O_3_ decomposition on the catalyst surface. Bicarbonate may also scavenge the HO• produced on surface. In contrast, TBA reacts with HO• in bulk solution. Then, the authors stated that the catalytic ozonation occurred primarily via HO• oxidation on the catalyst surface and in bulk solution. In addition, a portion of the dye degradation may be attributed to molecular ozone. The variations in COD removal during 10 sequential repeated tests were monitored and the activity was mostly maintained, for both raw and calcined samples.

Van and Trinh [166] studied the catalytic ozonation of organic compounds from landfill leachate focusing on the regression analysis of different operating parameters (pH, reaction time, amount of H_2_O_2_, ceramic raschig rings surface area, manganese ore amount). The manganese ore used in this study was mostly composed of MnO_2_ and SiO_2_ (about 75%), followed by Fe_2_O_3_ (11%), CaO (7%) and Al_2_O_3_ (5%). The average efficiencies of color, COD and TOC removal by ozone/manganese ore were about 6%, 20% and 14%, respectively, higher than ozone alone. The authors proposed a reaction mechanism based on the adsorption of the organic molecules onto the manganese ore surface, to be then oxidized by O_3_ and HO•.

Luo et al. [167] used a manganese modified silicate ore (MnSO by impregnation method) in the catalytic ozonation of ciprofloxacin (CIP). The characterization of the MnSO catalyst showed a homogeneously distributed MnOx (MnO_2_ and Mn_2_O_3_) over the surface of the silicate (SO) (13.35% *wt*/*wt* of Mn). The degradation rate constant of MnSO/O_3_ was 1.7 times and 3.3 times higher than those of SO/O_3_ and only O_3_, respectively. CIP removal was promoted at higher pH values (79.5% at pH 3.7 and 95.8% at pH 10.3), since under alkaline conditions more HO• was yielded. The addition of TBA significantly inhibited the reaction which indicated a mechanism based on the generation of hydroxyl radicals. The CIP removal was attributed to the synergistic effect of reagents’ adsorption, single ozone oxidation and HO• oxidation. Although slight leaching of manganese ions was found (0.069–0.097 mg/L), the catalytic capacity of MnSO was hardly affected.

### 4.4. Others

Among natural catalysts, several authors have used rocks or soils, which have a more complex composition, such as mixtures of different oxides, clays or organic matter. A brief description is presented below, and operating conditions and efficiency outcomes are presented in Table 4.

Yuan et al. [65] used pumice, a porous natural glass formed from volcanic activity with a relatively high concentration of silica, aluminum and iron. This material has been tested as a photocatalyst [168], a catalyst in the Fenton-like reaction [169] and as an adsorbent [170] in different water treatment processes. In this particular study, pumice was tested as a catalyst in the ozonation of trace concentrations of *p*-chloronitrobenzene (*p-*CNB). This model compound was chosen since it scarcely reacts with molecular ozone. The pumice selected presented a composition (XRF analysis) mainly centered on SiO_2_ (49.79%) and Al_2_O_3_ (15.6%) followed by Fe_2_O_3_ (9.15%), K_2_O (5.31%), MgO (4.15%), Na_2_O (4.05%) and TiO_2_ (3.13%). The material showed a low surface area (1.805 m^2^/g) and characterization by the saturated deprotonation method showed 0.27 mmol/g of surface hydroxyl groups, which are thought to be the responsible active sites. The presence of the catalyst enhanced the mineralization of the pollutant, with negligible adsorption of *p*CNB. In addition, the decomposition rate of aqueous ozone increased 1.374-fold in the presence of pumice. The authors performed experiments to determine HO• formation using the spin-trapping/EPR technique. Results suggested that in both ozonation alone and pumice-catalyzed ozonation, *p-*CNB was primarily oxidized by HO• in aqueous solution, under the operating conditions chosen (pH 6.86). Results also suggested that the presence of pumice generated higher HO• concentrations. This is in agreement with the outcomes obtained in the experiments performed in the presence of the radical scavenger TBA, which remarkably decreased the removal efficiency in the catalytic experiment, compared to the single ozonation test. The degradation efficiency was greatly enhanced by increasing pH from 3.01 to 8.93 in single and catalyzed ozonation experiments. The catalytic activity of the material was superior at pH near the pH_pzc_ value, where the relative HO• concentration was much higher than in single ozonation. The stability of the material was evaluated over 10 cycles, and it retained its catalytic activity with residual iron leaching.

Yuan et al. [171] also tested a modified pumice, Fe/pumice, as a catalyst in the ozonation of *p*-chloronitrobenzene (*p*-CNB). The catalyst was prepared by impregnation with Fe(NO_3_)_3_ and NaOH, then washed until constant pH and dried at 60 °C for 12 h. XRF analysis revealed a composition of O (45.8%), Si (20.5%), Fe (13.8%), Al (7.6%), Ca (4.5%), Na (3.0%), Mg (1.3%) and K (1.6%). The loading of Fe resulted in the growth of a new crystalline α-FeOOH phase and according to FTIR spectra the introduction of α-FeOOH significantly increased the height and width of surface hydroxyl groups peaks. The BET surface area increased from 0.12 to 33.5 m^2^/g, presenting a mesoporous type IV isotherm. The loading of α-FeOOH slightly increased the pH_PZC_ from 6.12 to 6.37 and the concentration of surface hydroxyl groups increased from 0.33 to 0.58 mmol/g. Fe/pumice enhanced the removal efficiency of *p*-CNB, the utilization efficiency of ozone, and the production of hydroxyl radical (HO•) relative to pumice alone during catalytic ozonation. The authors observed that SiO_2_ did not promote the decomposition of ozone into HO•. The zero-charging of the catalyst surface benefited HO• generation. The ozone adsorption capacity was higher for Fe/pumice, despite its lower Si/metal oxide ratio. Then, adsorption may have been dominated by the mesoporous structure of the modified sample. According to the authors, the modification of pumice increased the number of surface hydroxyl groups and the degree of ozone adsorption (mesopority as a key factor) on the catalyst surface, resulting in enhanced collision probability between surface hydroxyl groups on Fe/pumice metal oxides and ozone molecules, accelerating HO• formation. The stability of the modified material was evaluated over five cycles and it retained its catalytic activity with residual iron leaching. The homogeneous contribution of leached iron was insignificant relative to sole ozonation.

**Table 4:** Other minerals as catalysts in the ozonation of organic pollutants.

Catalyst	Pollutant	Operating Conditions	Reaction Outcomes	Ref.
Pumice	*p*-Chloronitrobenzene (p-CNB)	Semibatch reactor 1.2 L, total applied O_3_ 0.6 mg/L, 23 °C, 1 L of 100 µg/L *p*-CNB, catalyst load 1 g/L, 20 min, pH_0_ 6.86.	*p*-CNB Conversion: O_3_ alone: 54%, pumice + O_3_: 84.3%. Adsorption: 3.9%. Negligible leaching (Si, Al, Fe, Mg, Ti, K, Na).	[65]
Fe-pumice	*p*-Chloronitrobenzene (p-CNB)	Batch reactor 1 L, O_3_ 0.9 mg/L in liquid phase, 25 °C, 1 L of 100 µg/L *p*-CNB, catalyst load 0.5 g/L, 15 min, pH 6.	*p*-CNB Conversion: O_3_ alone: 40%, pumice+O_3_: 76%, Fe-pumice + O_3_: 91%. Adsorption < 5.5%. Fe leaching 2.1 µg/L.	[171]
Iron silicate-loaded pumice	Diclofenac (DCF)	Semibatch reactor, gas flow rate 1 L/min, O_3_ dose 5.52 mg/L, 25 °C, 0.5 L of 29.6 mg/L DFC, catalyst load 0.8 g/L, 60 min, pH_0_ 7.	TOC Conversion: O_3_ alone: 32.3%, O_3_ + cat.: 73.3%. DFC Conversion: O_3_ alone: 100%, O_3_+cat.: 100%, O_2_ + cat.: 7.3%.	[67]
Volcanic rocks P1 and P2	Parabens: methyl (MP), ethyl (EP), propyl (PP), benzyl (BeP) and butylparaben (BuP)	Semibatch reactor 2 L, gas flow rate 0.2 L/min, transferred O_3_ dose 20 mg/L, 25 °C, parabens 10 mg/L, catalyst load 0.5 g/L, pH_0_ 3.5.	O_3_ alone Conversion: 28% (MP), 26% (PP), 44% (BeP), 26% (EP), 41% (BuP). P1+O_3_ Conversion: 72% (MP), 72% (PP), 85% (BeP), 68% (EP), 70% (BuP). P2+O_3_ Conversion: 62% (MP), 58% (PP), 70% (BeP), 58% (EP), 64% (BuP). Adsorption negligible. Leaching: 0.35 Al, 0.45 Fe, 0.15 Na and 0.69 mg/L Mg.	[172]
Volcanic rock	Parabens: methyl (MP), ethyl (EP), propyl (PP), benzyl (BeP) and butylparaben (BuP)	Semibatch reactor 2 L, gas flow rate 0.2 L/min, 25 °C, 2 L of mixture of parabens 10 mg/L each (total COD 90 mg/L), catalyst load 0.5 g/L, pH_0_ 5.2.	COD Conversion: O_3_ alone <15%, O_3_ + cat.: 37% (with transferred O_3_ dose of 55 mg/L). Adsorption negligible. Leaching: 0.13 Al, 0.21 Fe, 1.12 Na, 3.18 Ca and 0.2 mg/L Mg.	[173]
Sepiolite, volcanic rock and iron shavings (zerovalent iron (ZVI))	Simulated olive mill wastewater	Semibatch reactor 0.5 L, gas flow rate 0.5 L/min, O_3_ 20 g/Nm^3^ in gas phase, 20 °C, 0.5 L of 1211 mg/L COD, catalyst load 1 g/L, 120 min, pH 3.	COD Conversion: O_3_ alone: 29%, sepiolite+O_3_: 31%, Volcanic rock + O_3_: 37%, ZVI + O_3_: 60%. Adsorption negligible. Fe leaching: 400 mg/L (ZVI).	[174]
Volcanic sand (VS)	Benzothiazole (BT)	Differential circular flow reactor (20 °C): after reaching dissolved O_3_ saturation (125 µM, 20 °C) the gas flow (120 L/h) is closed, BT is added (222 µM) and begins liquid recirculation (1 L/min, 1 L) through the fixed bed (10 g/L cat., 19 mL).	BT Conversion at pH 2, 10 min: O_3_ alone: 35%, VS + O_3_: 70%, VS: 5%. BT Conversion at pH 7, 10 min: O_3_ alone: 74%, VS+O_3_: 93%.	[88]
Soil: Sand containing organic matter (S), baked sand (BS) and goethite (G)	p-Chlorobenzoic acid (p-CBA)	Batch reactor, O_3_ dosage 3 mg/L, 25 °C, 2.56 × 10^−3^ mM pCBA, catalyst load 50 g/L (S, BS) and 5 g/L (G), pH 5.6, 420 s.	*p*-CBA Conversion: BS+O_3_: 73.2%, S + O_3_: 86%, G+O_3_: 88%.	[175]
Natural Mackinawite	N,N-dimethylacetamide (DMAC)	Semibatch reactor 0.25 L, 30 °C, gas flow rate 0.3 L/min, O_3_ 50 mg/L in gas phase, catalyst load 3.5 g/L, 20 min, pH_0_ 6.8.	DMAC Conversion: O_3_ alone: 9.2%, catalytic O_3_: 96.6%. Adsorption 3%. Fe leaching: 209.2 mg/L.	[49]
Polonite^®^ (POL), Wollastonite (WOLL), zeolite (ZeoCat)	Contaminants of emerging concern (CECs: Atrazine (ATZ), ibuprofen (IBP), naproxen (NPX), and gemfibrozil (GBZ)) in Milli-Q (MQW) and simulated synthetic wastewater (SWW)	Semibatch reactor 1 L, gas flow rate 1 L/min, O_3_ 8 g/Nm^3^ in gas phase, room T, 0.8 L of SWW (pH 7.6) or MQW (pH 5–6) with 150 µg of CECs, catalyst load 25 g/L. Disinfection tests: SWW spiked with *E. coli* 2 × 10^5^ MPN/mL.	ATZ Conversion with a total ozone dosage (TOD) of 18 mg/L, in MQW: O_3_ alone: 50%, O_3_+POL: 100%, O_3_+WOLL: 44%, O_3_+ZeoCat: 46%. Adsorption < 13%. ATZ Conversion with TOD of 40 mg/L, in SWW: O_3_ alone: 87%, O_3_+POL: 79%, O_3_ + WOLL: 65%, O_3_ + ZeoCat: 79%. TOD to reach disinfection criteria in SWW: POL or ZeoCat + O_3_: 34–39 mg/L, O_3_ alone: 38–49 mg/L.	[176]
Tourmaline (TOU)	Atrazine (ATZ)	Batch reactor 0.25 L, O_3_ 3 mg/L in liquid phase, 5 °C, 5 µM ATZ, catalyst load 1 g/L, 30 min, pH_0_ 7.	ATZ Conversion: O_3_ alone: 28.8%, O_3_ + TOU: 100%. Adsorption < 3%. Leaching: Fe (3.2–4.6 µg/L) and Al (6.8–5.4 µg/L)	[177]
Calcined Zeolite, Talc and Kaolin	p-Chlorobenzoic acid (p-CBA)	Batch reactor, O_3_ 2 mg/L in liquid phase, 23 °C, 4 µM p-CBA, catalyst load 0.5 g/L, 2 min, pH_0_ 7.	p-CBA Conversion: O_3_ alone: 94.5%, O_3_ + Zeolite: 99.5%, O_3_ + Kaolin: 95%, O_3_ + Talc: 98.7%. Adsorption negligible.	[178]
Brucite (Mg(OH))	Azo dye active brilliant red (X-3B)	Semibatch reactor, O_3_ flow rate 0.3 mg/min, 20 °C, 0.05 L of 500 mg/L X-3B, catalyst load 0.5 g, 15 min.	COD Conversion: O_3_ alone: 9%, Mg(OH) + O_3_: 33%. X-3B Conversion: O_3_ alone: 47%, Mg(OH) + O_3_: 89%. Adsorption 4%.	[179]
Brucite (Mg(OH)) and Magnesia (MgO)	Phenol (Ph)	Semibatch reactor, gas flow rate 5 mL/min, O_3_ 0.36 mg/min, 25 °C, 0.1 L of 100 mg/L Ph, catalyst load 5 g/L, 60 min, pH_0_ = 6.35 (O_3_), 10.18 (Mg(OH)), 10.8 (MgO).	COD Conversion: O_3_ alone: 38%, Mg(OH) + O_3_: 58%, MgO + O_3_: 90%. Adsorption negligible	[180]
Galena (PbS)	O-isopropyl-ethylthionocarbamate (IPETC)	Semibatch reactor, O_3_ dosage 2.065 mg/(min L), 2 L of 100 mg/L IPETC, catalyst load 0.75 g/L, 20 min, pH 10.	IPETC Conversion: O_3_ alone: 50%, PbS + O_3_: 90%. Adsorption ≈ 10% Leached Pb 14.9–23.2 mg/L (180 min) and ≈1 mg/L (20 min).	[181]
CuS	C. I. Reactive Blue 5 (RB-5)	Semibatch reactor, O_3_ 1 wt.%, gas flow rate 1.23 SLPM, 21–23 °C, 0.25 L of 1 g/L RB-5, catalyst load 1.2 g/L, 10 min.	Color Conversion: O_3_ alone: 57%, CuS + O_3_: 90%.	[182]
CuS	Acid Red-151 (AR-151), Remazol Brilliant Blue-R (RBBR), Reactive Black-5 (RB-5)	Semibatch reactor, O_3_ dosage 115 mg/(min L), 100 mg/L dye, catalyst load 0.1 g/L, 80 min, pH 3, 7, 10.	TOC Conversion pH 3: O_3_ alone: 25% (AR-151), 18% (RBBR), 24% (RB-5); CuS + O_3_: 40% (AR-151), 38% (RBBR), 40% (RB-5). Cu leached 27.7 mg/L TOC Conversion pH 7: O_3_ alone: 54% (AR-151), 48% (RBBR), 43% (RB-5); CuS+O_3_: 86% (AR-151), 83% (RBBR), 75% (RB-5). Cu leached 7 mg/L TOC Conversion pH 10: O_3_ alone: 63% (AR-151), 66% (RBBR), 79% (RB-5); CuS + O_3_: 95% (AR-151), 93% (RBBR), 86% (RB-5). Cu leached 4 mg/L.	[183]

Gao et al. [67] prepared an iron silicate-loaded pumice (FSO/PMC) for the catalytic ozonation of diclofenac (DCF). Na_2_SiO_3_ and Fe(NO_3_)_3_ were used as precursors in the preparation of the FSO/PMC sample. The polymerization of iron-silicon oxide on the surface of pumice was promoted by the dropwise addition of ammonium hydroxide, under mechanical stirring at ambient temperature. The sample was washed until constant conductivity and dried at 80 °C (24 h). The results showed that the FSO/PMC catalytic ozonation process significantly improved the DCF mineralization from 32.3% (sole-ozonation) to 73.3%. The decomposition of O_3_ into HO• occurred in both processes. However, the presence of FSO/PMC increased the HO• yield, which was verified through experiments in the presence of a radical scavenger (NaHSO_3_) and using the ESR spin-trap technique. In addition, the presence of FSO/PMC apparently accelerated the transfer of ozone from gas to the liquid phase, since the catalyst could act as a reservoir to temporarily store the soluble ozone. The adsorption of DFC was insignificant, but the adsorption of reaction intermediates was much higher. The authors proposed that the accumulation (i.e., adsorption) of various intermediates on the surface of the catalyst could increase the contact probability with HO• radicals. In addition, the authors corroborated that the higher TOC removal observed in the catalytic ozonation of DCF was dominated by the effective catalytic reaction, rather than the combination of FSO/PMC adsorption and sole-ozonation oxidation. The surface hydroxyl groups were proposed to be the active sites, showing higher activity at pH near the pH_PZC_ (7.21). The authors explained this behavior through the electrophilic and nucleophilic nature of the ozone molecule (resonance structure), which can simultaneously interact with the H (electrophilic) and O (nucleophilic) atoms of surface hydroxyl groups, forming a ring, which breaks down with the formation of O_2_•^−^ and then HO•. However, under acid and alkali conditions, the H atom is either protonated or deprotonated in aqueous solution, reducing the probability of interaction between ozone and hydroxyl groups.

Gomes et al. [172] studied the catalytic and photocatalytic ozonation of a mixture of parabens employing volcanic rocks collected in São Miguel (Azores, Portugal). The samples used in this study were: pumice (P2) and a material morphologically similar to pumice but with higher density (P1). The materials were washed (tap and distilled water) and dried at 105 °C for 24 h. XRD analysis of P1 showed peaks corresponding to augite and diopside, while P2 resulted in a kind of pumice due to the low density and high porosity. The BET analysis revealed surface areas of 28.3 and 2.98 m^2^/g for P1 and P2, respectively, and the samples presented similar pH_PZC_ (5.7 for P1 and 5.5 for P2). The catalytic ozonation with volcanic rocks allowed total paraben degradation using a transferred ozone dose threefold lower than the amount corresponding to single ozonation. In addition, adsorption was negligible under the pH values studied. The best performance was shown by P1, which was be connected to the higher surface area. According to EDX results and elemental analysis, both samples contained metal oxides, such as Al_2_O_3_ and Fe_2_O_3_, species which are known to promote ozone decomposition.

Iron leached was only 0.45 mg/L and the homogeneous contribution was neglected. Neutral and basic conditions enhanced the catalytic ozonation process and a mechanism based on radicals was confirmed through experiments with iso-propanol as a radical scavenger. This was also established through the analysis of by-products. UVA radiation showed no relevant effect on catalytic ozonation, possibly due to the lower number of semiconductors available on composition.

Gomes et al. [173] also used a volcanic rock as a catalyst in the ozonation of a mixture of parabens but focused on the toxicity evolution of the aqueous samples. As before, the volcanic rock was collected in São Miguel (Azores, Portugal), with a composition centered on silica and aluminum, with augite and diopside as main minerals, and a specific surface area of 28.3 m^2^/g.

The adsorption of parabens on the catalyst was negligible and they were totally removed by both single and catalytic ozonation. However, the toxicity of the samples resulting from both treatments was generally high. The catalytic ozonation allowed reducing the amount of ozone (about 3-fold) required for total removal of parabens. However, the resulting treated solution was more toxic than the sample taken at the endpoint of the single ozonation treatment. This suggests that the higher amount of ozone used in single ozonation allowed the elimination of toxic by-products.

Martins et al. [174] tested red volcanic rock, sepiolite, and iron shavings (zero valent iron, ZVI) as catalysts in the ozonation of olive mill wastewater. The sepiolite and volcanic rock were commercially available, whereas iron shavings were collected from a metallurgic industry. Sepiolite and volcanic rock were mostly composed of SiO_2_ with traces of Fe, Na, Ca and Mg in volcanic rock and Mg, Ca, Fe, H and O in sepiolite. ZVI was mostly composed of Fe. XRD characterization showed the presence of ferritic steel in ZVI. Magnesium silicate hydroxide hydrate was identified in sepiolite. The volcanic rock presented a more complex mineralogy, with poorly defined peaks of low intensity, showing the presence of hematite, pyroxene, quartz and feldspar. The red volcanic rock (3.49 ± 0.02 m^2^/g) and ZVI (1.14 ± 0.04 m^2^/g) presented low specific surface areas compared to sepiolite (226.65 ± 7.00 m^2^/g). ZVI showed the highest oxidation efficiency and the other materials showed COD removal values close to those obtained in the non-catalytic system. The higher efficiency of ZVI was related to its higher metallic load. The iron leached concentration observed was quite high, 400 mg/L. Therefore, a separation step such as precipitation was recommended. The higher efficiency observed at pH 3 was attributed to the stronger oxidation of ZVI into ferrous and ferric iron, species that have shown activity in the catalytic ozonation of several organic compounds. Under acidic conditions, Fe^0^ may be oxidized into Fe^2+^, which is able to react with dissolved ozone to produce hydroxyl radicals. In addition, the presence of Fe^0^ is known to facilitate the reduction of Fe^3+^ into Fe^2+^. At high pH values, the precipitation of iron as hydroxides reduced the catalytic activity. The stability of the material was evaluated over four cycles. There was an important activity decay from the first to second test, then ZVI maintained its activity for the remaining trials. The loss of activity was related to the reduced iron leaching after each cycle or the formation of iron precipitates in the solid, that may have limited leaching and surface reactions. Regarding the reaction mechanism, experiments performed in the presence of radical scavengers (sodium carbonate and TBA) suggested a degradation mechanism based on radicals.

Valdés et al. [88] studied the catalytic ozonation of benzothiazole (BT) using volcanic sand (VS) as a catalytic material. This non-porous material (with pH_pzc_ 6.8) was collected from the Chillán volcano (Bío-Bío, Chile) and it was only sieved, washed (de-ionized water) and dried (105 °C, 24 h). The material was mostly composed (XRF analysis) of SiO_2_ (63.67%), Al_2_O_3_ (13.92%) and Fe_2_O_3_ (4.65%), with a minor contribution of Na, K, Ca, Mg, Ti, P and Mn. First, the authors studied O_3_ decomposition in aqueous phase in the presence of acetic acid as a radical scavenger, at different pH values. At pH 2, the presence of acetic acid led to 55% and 20% decreases in ozone decay rates, in the presence and absence of VS. At pH 8, the O_3_ decomposition rate without VS was inhibited by 54%. However, when VS was used in the presence of acetate ions, rate constants were not significantly affected. According to the authors, these findings would suggest that ozone decomposition reactions mainly occur on the mineral surface, not affected by the presence of radical scavengers. At pH > pH_PZC_, the increase in aqueous ozone decay in the presence of volcanic sand could be related to the interaction between ozone and strong Lewis acid centers on the metal oxide surface sites. The authors also studied the BT decomposition, which was enhanced by the presence of VS. In this case, the influence of TBA as a radical scavenger was evaluated (pH 2) in single and catalytic ozonation experiments. In both cases, a decrease in BT oxidation was observed when TBA was present. However, this effect was attenuated in the presence of VS. The authors proposed a reaction mechanism characterized by a combination of competing homogeneous and heterogeneous reactions: a direct reaction of molecular ozone and an indirect reaction involving non-selective free radicals, where strong Lewis acid sites on the volcanic sand surface could act as initiators and/or promoters of radical chain reactions.

Lim et al. [175] studied the catalytic decomposition of ozone on a sand and iron surface. The authors used two samples of Jumunjin sand (Korea): sand (S) with 0.12% wt. of soil organic matter (SOM) and baked sand (BS), which was calcined at 550 °C for 24 h. The raw material was mostly composed (XRF analysis) of SiO_2_ (90.41%), Al_2_O_3_ (5.48%) and Fe_2_O_3_ (0.12%), with a minor contribution of Na, K, Ca, Mg, Ti, P and Mn. To verify the formation of hydroxyl radicals, *para*-chlorobenzoic acid (*p*-CBA) was selected as a probe molecule. The generation of hydroxyl radicals was demonstrated and was closely related to metal oxides (MO) in BS as well as soil organic matter (SOM) in S. Ozone decomposition and the reaction between hydroxyl radicals and *p*-CBA appeared to be independent of any change in pH. Metal oxides (MO) in the soil’s surface were considered to have a relatively faster reaction rate with ozone and provided more favorable reactive sites to generate higher amounts of HO• than SOM. Following this trend, the authors tested synthetic goethite and observed that even at one-tenth of the concentration of the sands, a goethite-induced catalytic reaction outfitted the removal rate of *p*-CBA among all the soils tested.

Peng et al. [49] used natural mackinawite (NM) as a catalyst in the ozonation of N, N-dimethylacetamide (DMAC). The authors purchased micro-sized natural mackinawite (NM) powder, mainly composed of iron sulfide (FeS > 85%). NM was compared to zero-valent iron and synthetic FeS. The degradation efficiency of DMAC in the NM/O_3_ process (i.e., 95.4%) was much higher than that with (ZVI)/O_3_ (i.e., 46.1%) or synthetic FeS/O_3_ (i.e., 68.6%). This phenomenon was attributed to the influence of other compounds in NM composition. Regarding the pH effect, the results showed that DMAC degradation in the O_3_ alone system increased as pH increased, with the highest DMAC conversion of 17.0%, at pH 10.0. However, in the catalytic system, degradation efficiency decreased at pH 9 or 10, indicating that the presence of OH^−^ ions was not a determinant for the catalytic activity. The authors observed that pH = pH_pzc_ was favorable for the catalytic performance, suggesting that zero charged surface hydroxyl groups were the main factor responsible for DMAC degradation. In order to determine the effect of different ROS on DMAC removal, different scavengers were employed: tert-buty alcohol (TBA) and phosphate (H_2_PO_4_^−^) for HO•, and superoxide dismutase (SOD) and catalase (CAT) for O_2_•^−^ and H_2_O_2_. Results indicated that the extraordinary efficiency for DMAC degradation was mainly caused by HO•. The stability of the material was tested over three cycle experiments and DMAC conversion slightly decreased after each use. This was attributed to the slight oxidation of the catalyst surface. Iron leaching was evaluated and concentrations between 262.2 and 164.2 mg/L were detected in the pH range from 3 to 10. However, the effect of the leached iron was not a dominant factor for the oxidation efficiency.

Kolosov et al. [176] compared different novel commercial materials: Polonite^®^, wollastonite, zeolite, TiO_2_-Al_2_O_3_ (8%/92%) and AL-1010S (AlO_2_-based) in the catalytic ozonation of contaminants of emerging concern (CECs: Atrazine (ATZ), ibuprofen (IBP), naproxen (NPX) and gemfibrozil (GBZ)) in Milli-Q (MQW) and simulated synthetic wastewater (SWW) (to mimic municipal secondary effluent). Polonite^®^, wollastonite and the zeolite selected are natural materials, mined and commercialized in different areas of the world. Polonite^®^ is a product developed by Ecofiltration Nordic AB, obtained by high temperature heating of opoka, a natural calcium-silicate mineral, mined in southeastern Europe (Poland) and western Russia. The composition of Polonite^®^ includes: SiO_2_ (40% by weight), CaO (40%), Al_2_O_3_ (6%), Fe_2_O_3_ (2%), K_2_O (1%), MgO (1%), and other metal oxides such as TiO_2_, MnO_2_, P_2_O_3_. Wollastonite, a calcium silicate mineral, was provided by Canadian Wollastonite, Kingston, Ontario. It usually occurs as a common constituent of a thermally metamorphosed impure limestone. The typical composition of wollastonite consists of CaO (28%), SiO_2_ (49%), Al_2_O_3_ (10%), Fe_2_O_3_ (4%), K_2_O (5%), MgO (2%), and a small fraction of MnO_2_, TiO_2_ (% by weight). The zeolite, a silicate-based material containing alumina, was provided by ZeoCat Soluciones Ecológicas S.L.U, Spain. The authors evaluated the pollutant’s abatement and disinfection (*E. coli* removal) in different water matrices. Zeolite and wollastonite did not promote disinfection and CECs removal; TiO_2_-Al_2_O_3_ and AL-1010S showed improvements in both parameters, but to a lesser extent in SWW than in Milli-Q water; and Polonite^®^ had the most important effect on lowering the total ozone dosage (TOD) required for disinfection but scarcely impacted CECs removal. The reaction pH value changed differently for each catalyst: for zeolite decreased to 4.5, for wollastonite and TiO_2_-Al_2_O_3_ shifted to values between 6 and 7, while in the presence of Polonite^®^, pH increased above 10, which was attributed to the caustic nature of the mineral and reactive CaO phase. As previously mentioned, the increase in pH creates favorable conditions for the formation of HO• radicals. However, according to the experiments with adjusted initial pH, the increase in pH could not alone explain the higher removals obtained with Polonite^®^. Experiments with pCBA indicated that HO• played a significant role in the catalytic and noncatalytic treatments, but with greater contribution in the catalytic ones. Since zeolite and wollastonite showed low activity in the operating conditions studied, the kinetic and stability studies were centered on Polonite^®^, AL-1010S and TiO_2_-Al_2_O_3_. The catalysts were successfully reused over four consecutive cycles of 6 h in a continuous ozonation system. According to the results obtained, Polonite^®^, AL-1010S and TiO_2_-Al_2_O_3_ can provide mechanisms to lower the ozone dose required to reach disinfection.

In a following study, Kolosov and Yargeau [184] evaluated the impact of some operating conditions (ratio of ozone feed concentration to catalyst load) and wastewater characteristics (COD and nitrite) on the disinfection and removal of CECs during catalytic ozonation using Polonite^®^ and AL-1010S. The presence of the catalysts enhanced disinfection and atrazine removal in SWW, by making the treatment performance less sensitive to increments in COD and nitrite.

Wang et al. [177] used tourmaline in the catalytic ozonation of atrazine. XRD, FESEM, EDX, TEM, FTIR, pyridine-FTIR and XPS analyses confirmed that tourmaline was mainly composed of Ca, B, Na, Al, Si, Fe and O, with a surface area of 8.68 m^2^ g^−1^. Moreover, surface hydroxyl groups attached to metallic ions were the main active sites for ozone adsorption and decomposition. EPR measurements and experiments with TBA confirmed a catalytic mechanism based on the generation of radicals. The material was used in five recycling runs, confirming its stability and reusability. Al and Fe leaching was far below the limitations of the drinking water quality standard.

Psaltou et al. [178] studied the effect of thermal treatments on three inexpensive natural materials: zeolite, talc and kaolin, to be used in the catalytic ozonation of p-CBA. The zeolite and talc were thermally treated at various temperatures up to 800 °C, while kaolin was only calcined at 600 °C. The specific surface area of the materials was rather low, e.g., 21, 10.5 and 13 m^2^/g for zeolite, talc and kaolin, respectively. The calcination treatment caused an increase in the pH_PZC_ value and hydrophobicity of the materials, due to the dihydroxylation of the surface. According to previous studies, ozone molecules tend to approach neutrally charged and non-polar surfaces more effectively, enhancing their decomposition into hydroxyl radicals. This explained the different catalytic activity observed for the studied samples. Zeolite and talc, presenting pH_PZC_ of 7.2 and 6.5, respectively, showed higher catalytic activity after thermal treatment, while kaolin with a pH_PZC_ of 3.1, showed zero to moderate catalytic efficiency. This was connected to its strongly negatively charged surface in the neutral pH range, where most of the experiments were performed. Experiments with TBA confirmed the presence of radicals in the catalytic mechanism.

Dong et al. [179] used natural brucite (Mg(OH)_2_) to catalyze the ozonation of azo dye Active Brilliant Red X-3B. According to TBA experiments, the catalytic ozonation followed a direct oxidization mechanism by ozone molecules, which was enhanced by the homogeneous catalytic effect of hydroxyl anions generated from Mg(OH)_2_ dissolution.

In a following study, He et al. [180] used natural brucite (Mg(OH)) and magnesia (MgO) in the catalytic ozonation of phenol. The natural sample was calcined at 450 °C for 6 h, to obtain magnesia (MgO). Both samples enhanced phenol and COD removal and it was related to the pH variations generated by the materials. In the catalytic ozonation process with brucite, the pH value varied between 10.18 and 8.52. While in the magnesia system the pH value was maintained nearly around 10.80. Under the alkaline environment of brucite, ozone showed a higher reactivity towards the negatively charged phenol and decomposed products due to its electrophilic characteristics. In this case, the direct oxidation mechanism with molecular ozone played the dominant role. In the case of magnesia, the enhancement by hydroxyl radical generation was more important.

Metal sulfides, abundant in the earth’s crust, have been tested as catalytic materials in the ozonation of different organic pollutants. Fu et al. [181] studied the use of galena (PbS), a common sulfide mineral in many ore deposits, as a catalyst in the ozonation of *o*-isopropyl-*N*-ethylthionocarbamate (IPETC), a typical sulfide flotation reagent. The authors described that several fine sulfide minerals (e.g., galena, sphalerite, chalcopyrite, pyrite and arsenopyrite) may remain in flotation effluents, which could affect the efficiency of the catalytic ozonation process of this type of polluted water. In this case, galena, usually found in flotation effluents, was chosen as a mineral catalyst with a careful evaluation of its dissolution behavior, due to the high toxicity of Pb(II) ions. The studies were mostly performed under basic pH since the pH of flotation effluents usually ranges from neutral to alkaline. Best results were obtained at pH 10, with un-protonated galena (pH_PZC_ 2.8). Both the radical scavenger test (TBA) and equilibrium ozone concentration measurements suggested a catalytic mechanism based on radicals. The release of toxic Pb(II) ions was enhanced at neutral pH compared to alkaline pH. The formation of PbCO_3_ and PbSO_4_ on galena’s surface after catalytic ozonation was confirmed by XPS and FTIR analysis. Due to the important levels of Pb(II) leaching, the authors recommend a further removal of released heavy metal ions after the ozonation.

Yong et al. [182] performed a series of preliminary tests to select the most viable catalyst for C.I. Reactive Blue 5 ozonation, covering CuS, TiO_2_, ZnO, Al_2_O_3_, NiO, Cr_2_O_3_, MnO_2_ and CuO. Among the materials tested, only CuS accelerated dye discoloration. The efficacy of the ozonation process was higher at low pH and tests performed with TBA revealed a negligible participation of radicals. Then, the authors suggested that discoloration might occur by oxidation with molecular ozone and surface-bound oxygen instead of free radicals.

Pirgalıoğlu and Özbelge [183] studied the catalytic ozonation of single dye solutions with CuS. In this case, the homogeneous contribution was carefully evaluated, and the authors observed that the higher mineralization levels obtained were caused by the activity of dissolved copper ions, which enhanced the decomposition of ozone into hydroxyl radicals. According to the characterization results (XRD and EDX) the composition and chemical structure of the CuS catalyst did not change during the ozonation process. Only some amount of solid CuS was oxidized by ozone and passed into the solution as ions.

## 5. Conclusions

The use of cheap and widely available natural materials is a promising option that would enhance the application of ozone in a cost-effective water treatment process. Hence, this review presented a detailed description of outcomes obtained using natural materials (mostly clay, zeolites and oxides) as catalysts in the ozonation of organic pollutants. The structural characteristics that justify the selection of these natural materials for catalytic purposes were described, together with results referring to the degradation efficiency of organic pollutants and reaction mechanism observed. The preliminary results obtained are promising but arise from laboratory-scale experiments that often exclude the complexity of real wastewater matrices and do not optimize all the operating parameters, such as the ozone dose. The studies are mostly centered on elucidating the catalytic mechanism with a probe molecule.

The reported reaction mechanisms of the heterogeneous catalytic ozonation process and its advantages were described, with emphasis on the methodology employed to infer the reaction mechanism (i.e., radical scavengers, probe molecules, ESR technique), and highlighting the importance of catalyst characterization and well-designed experiments including proper blank tests.

The effects of operating parameters (pH, ozone and catalyst dose, temperature) on the evaluation of the reaction mechanism and degradation efficiency outcomes were emphasized.

Despite the large number of studies focused on elucidating the catalytic ozonation mechanism, there is still a lack of understanding. Contradictory results were obtained with materials of similar characteristics or with the same material and pollutants of different nature. Only few studies present a cost evaluation of the ozonation process, which depends on the objective of the treatment, and the type, volume and concentration of the effluent.

The vast majority of studies were performed with model pollutants in a lab scale. However, in order to promote the industrial application of the catalytic ozonation process, more studies centered on the treatment of real effluents at a pilot scale are required, with evaluation of stability and reusability of the catalytic materials and toxicity of the treated water.

Due to availability and lower cost, it would be easier to scale up the use of these natural materials. However, to achieve this, their efficiency and stability should be further analyzed in more realistic scenarios. On the other hand, an exhaustive characterization of the materials should be carried out to guarantee a consistent composition and avoid batch-to-batch variations as well as the presence of impurities that may interfere with the treatment or be a source of secondary contamination.

Cost evaluation and degradation efficiency results should be compared with the outcomes obtained with more sophisticated and expensive catalysts, in order to decide whether the natural mineral catalysts are an economically viable option, more ecological and cost-effective.

## Data Availability

Not applicable.
